# Comparative effectiveness of beta-interferons and glatiramer acetate for relapsing-remitting multiple sclerosis: systematic review and network meta-analysis of trials including recommended dosages

**DOI:** 10.1186/s12883-018-1162-9

**Published:** 2018-10-03

**Authors:** G. J. Melendez-Torres, Xavier Armoiry, Rachel Court, Jacoby Patterson, Alan Kan, Peter Auguste, Jason Madan, Carl Counsell, Olga Ciccarelli, Aileen Clarke

**Affiliations:** 1 Warwick Evidence, Warwick Medical School, University of Warwick, Coventry, CV4 7AL UK; 2 Independent research consultant, Windsor, UK; 3 Institute of Applied Health Sciences, University of Aberdeen, Aberdeen, UK; 4 Queen Square Multiple Sclerosis Centre, University College London Institute of Neurology, London, UK; 5 National Institute for Health Research University College London Hospitals Biomedical Research Centre, London, UK

**Keywords:** Multiple sclerosis, Clinically isolated syndrome, Beta-interferon, Glatiramer acetate, Systematic review, Economic evaluation

## Abstract

**Background:**

We systematically reviewed the comparative effectiveness of injectable beta-interferons (IFN-β) and glatiramer acetate (GA) on annualised relapse rate (ARR), progression and discontinuation due to adverse events (AEs) in RRMS, using evidence from within the drugs’ recommended dosages.

**Methods:**

We updated prior comprehensive reviews, checked references of included studies, contacted experts in the field, and screened websites for relevant publications to locate randomised trials of IFN-β and GA with recommended dosages in RRMS populations, compared against placebo or other recommended dosages. Abstracts were screened and assessed for inclusion in duplicate and independently. Studies were appraised using the Cochrane risk of bias tool. Rate ratios for ARR, hazard ratios for time to progression, and risk ratios for discontinuation due to AEs were synthesised in separate models using random effects network meta-analysis.

**Results:**

We identified 24 studies reported in 42 publications. Most studies were at high risk of bias in at least one domain. All drugs had a beneficial effect on ARR as compared to placebo, but not compared to each other, and findings were robust to sensitivity analysis. We considered time to progression confirmed at 3 months and confirmed at 6 months in separate models; while both models suggested that the included drugs were effective, findings were not consistent between models. Discontinuation due to AEs did not appear to be different between drugs.

**Conclusions:**

Meta-analyses confirmed that IFN-β and GA reduce ARR and generally delay progression as defined in these trials, though there was no clear ‘winner’ across outcomes. Findings are additionally tempered by the high risk of bias across studies, and the use of an impairment/mobility scale to measure disease progression. Future research should consider more relevant measures of disability and, given that most trials have been short-term, consider a longitudinal approach to comparative effectiveness.

**Review registration:**

PROSPERO CRD42016043278.

**Electronic supplementary material:**

The online version of this article (10.1186/s12883-018-1162-9) contains supplementary material, which is available to authorized users.

## Background

Injectable beta-interferons (IFN-β) and glatiramer acetate (GA) are mainstays of first-line treatment for relapsing-remitting multiple sclerosis (RRMS), with the primary goals of reducing the rate of relapses and delaying disease progression. Newer therapies such as alemtuzumab yield greater effects in reducing relapse rate and slowing disease progression, and patients may prefer therapies such as dimethyl fumarate or teriflunomide because of their oral mode of administration. However, amongst other disease-modifying therapies (DMTs), IFN-β and GA both have well-established long-term safety profiles without the severe side effects presented by other drugs. While IFN-β and GA are not appropriate for aggressive forms of RRMS (i.e. highly active RRMS or rapidly evolving-severe RRMS), the Association of British Neurologists (ABN) classifies these as ‘drugs of moderate efficacy’ [1]. Beginning in 2017, an appraisal committee of the UK National Institute for Health and Care Excellence received evidence as part of its reconsideration of the clinical and cost effectiveness of IFN-β and GA for use in the UK National Health Service. The work presented here, the full record of which can be found at [2], draws from our report to this appraisal committee.

There are currently five licensed IFN-β drugs indicated for RRMS. These include: two IFN β-1a (Avonex® (Biogen, Cambridge, Massachusetts, USA), administered via intramuscular injection once weekly at a dose of 30 μg; and Rebif® (Merck, Darmstadt, Germany), administered via subcutaneous injection three times weekly at a dose of either 44 or 22 μg); one pegylated IFN β-1a (Plegridy® (Biogen, Cambridge, Massachusetts, USA), administered via subcutaneous injection every 2 weeks at a dose of 125 μg); and two equivalent IFN β-1b (Betaferon® (Bayer, Leverkusen, Germany) and Extavia® (Novartis, Bale, Switzerland), both administered via subcutaneous injection every other day at a dose of 250 μg). Moreover, there are two licensed formulations of GA (Copaxone® (Teva, Petah Tikva, Israel)), both administered via subcutaneous injection: one at a dose of 20 mg daily, and another at a dose of 40 mg three times weekly. The mechanisms by which either type of drug exerts its effects in patients with MS are not fully understood, but it is now thought that these drugs induce a broad immunomodulatory effect that modifies the immune processes responsible for the pathogenesis of MS.

Though several systematic reviews incorporating network meta-analyses (NMAs) have considered the comparative effectiveness of treatments for RRMS, these have considered doses that do not correspond to the marketing authorisation and thus are not relevant to clinical practice (Tramacere et al. [3], Filippini et al. [4]), excluded relevant doses within drugs’ marketing authorisations (Tolley et al. [5]), or included trials across differing severities of MS (Hadjigeorgiou et al. [6]). Our goal in this systematic review and NMA is to provide an up-to-date and consistent summary of the comparative effectiveness of IFN-β and GA on annualised relapse rate (ARR), disability progression and discontinuation due to adverse events (AEs) in RRMS, using evidence from within the drugs’ recommended dosages.

## Methods

This systematic review was part of a larger evidence synthesis project considering the effectiveness of treatments for several types of MS. Our protocol is registered on PROSPERO as CRD42016043278. The methods and results described here draw on our closely related work for the UK National Institute for Health and Care Excellence, the full report of which was provided to the National Institute for Health Research [2]. In the original protocol, we described that we would stratify comparisons by type of MS. Here, we report clinical effectiveness findings relating to RRMS specifically.

### Searches

We identified and examined past relevant systematic reviews, conducted update searches in multiple databases, checked references of included studies, contacted experts in the field, and screened websites for relevant publications. We undertook the main database searches in January and February 2016. These update searches were limited by date to the beginning of 2012 (the year the searches were undertaken for the last comprehensive systematic review and NMA by Filippini et al. [4]) onwards, although we included trials without regard to publication date. This review was chosen because of the breadth of its scope, search strategy and eligibility criteria. A full record of searches is provided in Additional file 1.

We included: a) randomised controlled trials published as full-text reports in English (as well as systematic reviews, or meta-analyses to enable reference checking), b) in people diagnosed with RRMS, c) where the intervention was one of the drugs used within indication at the recommended dosage according to the summary of product characteristics as authorised by the European Medicines Agency (EMA), and d) where the comparator was placebo or best supportive care without DMTs, or another of the interventions when used within indication. Included trials had patient populations primarily comprised of RRMS patients. Our primary outcomes were relapse frequency, disease progression, and discontinuation due to adverse events. Outcomes assessed were relapse rate, time to progression, or discontinuation due to adverse events as outcomes. Full exclusion criteria can be found in the review protocol.

### Study selection

First, two authors (XA and GJMT) independently examined relevant past systematic reviews (including Tramacere et al. [3], Filippini et al. [4], and Clerico et al. [7]) for studies meeting the inclusion criteria. We verified inclusion of these studies by examining their full text. For updated and new searches, we collected all retrieved records in a specialised database and removed duplicate records. We pilot-tested a screening form based on the predefined study inclusion and exclusion criteria. Subsequently, two reviewers (XA and GJMT) applied the inclusion/exclusion criteria and screened all identified bibliographic records on title/abstract and then using full texts. Any disagreements over eligibility were resolved through consensus or by a third party reviewer (AC). Reasons for exclusion of full text papers were documented.

### Appraisal and extraction

All primary studies were appraised using the Cochrane risk of bias assessment tool [8]. For all included studies, the relevant data were extracted independently by two reviewers using a data extraction form informed by the Centre for Reviews and Dissemination [9]. Extracted data were entered into summary evidence Tables. A sample data extraction form is available in Additional file 1. Uncertainty and/or any disagreements were cross-checked with recourse to a third reviewer where necessary and resolved by discussion.

### Meta-analysis

We undertook separate meta-analyses corresponding to each of our review outcomes. Data preparation methods to generate summary effect sizes for each study are detailed in Additional file 1.

First, for relapse frequency, we elected to meta-analyse rate ratios (RR) of relapses as an overall measure. This was the most commonly reported measure for relapse frequency. Where necessary, we converted arm-level data into rate ratios. Where studies presented different estimates for relapse frequency, we preferred estimates of protocol-defined, clinician-confirmed relapses over non-protocol-defined relapses or self-reported relapses.

Second, disease progression is frequently defined in clinical trials of DMTs in MS using the Expanded Disability Status Scale (EDSS), a scale which ranges from 0 to 10. While the EDSS is described as a disability scale (and thus, trials present this as disability progression), it is perhaps better understood as a scale measuring impairment and mobility. We used hazard ratios (HR) to examine differences between study arms in time to progression, where progression was confirmed at either 3 or 6 months after an initial signal (generally an increase in EDSS of 0.5 or 1.0 points). We separated estimates for progression confirmed at 3 months and confirmed at 6 months, as we could not establish whether measures were commensurate.

Third, we estimated models for discontinuation due to AEs, using risk ratios as a summary measure. We also estimated one model with studies closest to 24 months of follow-up. This was because risk ratios are time dependent and we could not reliably estimate person-years of follow-up in each arm across all studies to convert study-level estimates to rate ratios.

We pooled outcomes for each intervention-comparator contrast using random effects meta-analysis in Stata v14 and examined these pairwise meta-analyses for heterogeneity, measured as Cochran’s Q and I^2^. Subsequently, we used the package -network- [10] in Stata v14 to estimate network meta-analyses. We used a common heterogeneity model, where the between-studies variance is assumed equal across comparisons. After estimating a consistency model (i.e. where direct evidence for a contrast between two treatments is assumed to agree with indirect evidence for that contrast), we checked for inconsistency using an omnibus Wald test from a design-by-treatment interaction model and the side-splitting method to test for differences in the effectiveness estimates between direct and indirect evidence. Where evidence of inconsistency existed, we considered the direction of inconsistency. We also assessed transitivity conceptually by examining networks of evidence for imbalance of trial-level effect modifiers (e.g. sex, age and duration of MS diagnosis; date of trial publication), though we did not have enough studies on each comparison to undertake network meta-regression.

Lastly, we used a bootstrapping method to resample from our estimates of intervention effectiveness and develop probabilities of each treatment’s relative position to the others. We then used the surface under the cumulative ranking curve (SUCRA) to produce a unified ranking of treatments.

### Publication bias

We aimed to use funnel plots to examine studies for the presence of asymmetry, possibly due to publication bias, other reporting biases, heterogeneity or methodological inadequacies in included studies, in pairwise comparisons where there were more than 10 studies for an intervention-comparator contrast.

## Results

### Search results

**Fig. 1:**
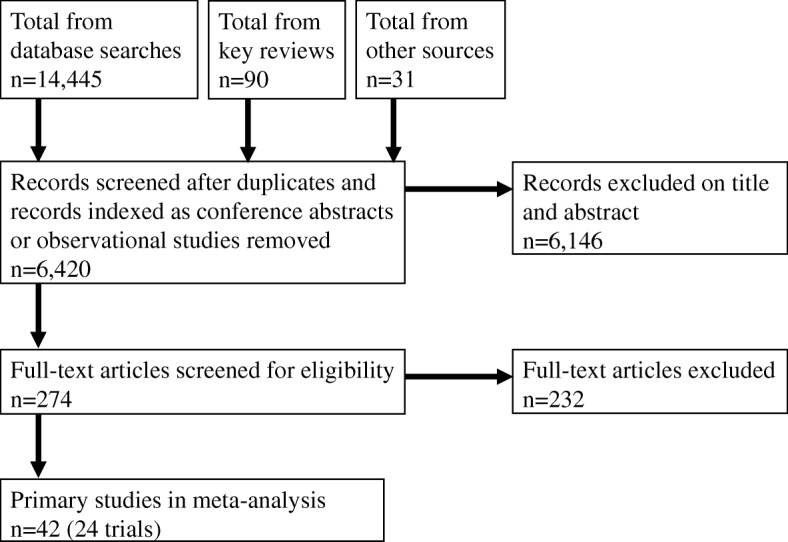
PRISMA flowchart

We identified 6420 potentially relevant records. We removed 6146 records which did not meet our inclusion criteria at title/abstract stage, leaving 274 records to be examined at full-text. Among these, we excluded 232, leading to 42 publications meeting our inclusion criteria and corresponding to 24 primary studies. Study selection is summarised in Fig. 1. Additional studies related to other MS phenotypes and are described in the full report of our work for the National Institute for Health and Care Excellence [2].

### Excluded studies

We excluded two trials in relevant populations and interventions because they did not present relevant outcomes (Schwartz 1997 [11]) or did not present outcomes in a form suitable for meta-analysis (Mokhber 2014 [12]). We also excluded one small trial with a mixed RRMS/SPMS population (REMAIN 2012 [13], RRMS *n* = 13) as treatment switching was explicitly allowed and data were not stratified by type of MS. Breakdown of studies by exclusion criterion is summarised in Additional file 2.

### Included studies

We included 24 trials published between 1987 and 2015. Included studies are detailed in Table 1. In total, 14 trials were placebo-controlled, of which three (BRAVO 2014 [14], CONFIRM 2012 [15] and Kappos 2011 [16]) principally aimed to test the effectiveness of a new agent against either IFN-β or GA alongside a placebo control. The remaining 10 trials only compared active drugs against each other. One trial (AVANTAGE 2014 [17]) reported only adverse events data. The modal follow-up was 24 months.

**Table 1:** Characteristics of included studies

Study ID MS type (diagnostic criteria)	Study details	Characteristics of participants at baseline	Intervention	Participants
ADVANCE 2014 RRMS (2005 McDonald criteria)	Country: USA, Belgium, Bulgaria, Canada, Chile, Colombia, Croatia, Czech Republic, Estonia, France, Georgia, Germany, Greece, India, Latvia, Mexico, Netherlands, New Zealand, Peru, Poland, Romania, Russian Federation, Serbia, Spain, Ukraine, United Kingdom. No. of countries: 26 Centres: 183 Study period: June 2009 and November 2011. Sponsor: Biogen Idec	Mean age: 36.5 (9.9) Mean sex: 71% female Race: 82% white EDSS Score: 2.5 Relapse rate: 1.6 within the previous 12 months, 2.6 within the previous 36 months Time from diagnosis of MS: 3.6 years Other clinical features of MS: Time from first MS symptoms: 6.6 years	Arm 1: pegylated IFN β-1a 125 μg SC every 2 weeks (Plegridy) Arm 2: Placebo	Randomised 512 arm 1 500 arm 2
AVANTAGE 2014 RRMS/CIS, diagnostic criteria unclear	Country: France No. of countries: 1 Centres: 61 Study period: March 2006–April 2008, 3 months follow up Sponsor: Bayer	Mean age: 38.7 Mean sex: 75% female Race: NA EDSS Score: *1.8 ± 1.3* Mean number of relapse rate: 2.1 ± 1.1 Time from diagnosis of MS: 3.3 (6.4) years Other clinical features of MS: NA	Arm 1: IFN β-1b 250 μg SC every other day (Betaferon) via Betaject Arm 2: IFN β-1b 250 μg SC every other day (Betaferon) via Betaject light Arm 3: IFN β-1a 44 SC three times weekly (Rebif) via Rebiject II	Included: 73 arm 1 79 arm 2 68 arm 3
BECOME 2009 RRMS/CIS (likely McDonald 2001 or 2005)	Country: USA No. of countries: 1 Centres: 2 Study period: Not specified, follow up over 2 years Sponsor: Bayer Schering pharma	Mean age: 36 Mean sex: 69% females Race: 52% white Median EDSS Score: *2* Relapse rate: 1.8 and 1.9 ARR Time from diagnosis of MS: between 0.9 and 1.2 Other clinical features of MS: 81% RRMS, 19% CIS; MSFC median 0.13	Arm 1: IFN β-1b 250 μg SC every other day (Betaferon) Arm 2: GA 20 mg SC daily (Copaxone)	Randomised 36 arm 1 39 arm 2
BEYOND 2009 RRMS (McDonald 2005)	Country: Not specified No. of countries: 26 Centres: 198 Study period: November, 2003, and June, 2005. Follow up between 2 and 3.5 years Sponsor: Bayer	Mean age 35.6 Mean sex: 69.4% female Race: 91.9% white EDSS Score: 2.33 Relapse rate: 1.6 relapses in last year Time from diagnosis of MS: 5.2 years Other clinical features of MS: 3.6 relapses previously; 70.6% had two or more relapses in past 2 years	Arm 1: IFN β-1b 250 μg SC every other day (Betaferon) Arm 2: GA 20 mg SC daily (Copaxone)	Randomised 897 arm 1 448 arm 2
Bornstein 1987 RRMS (Poser)	Country: USA No. of countries: 1 Centres: Not specified Study period: Not specified, follow up over 2 years Sponsor: public (grant from the National Institute of Neurological and Communicative Disorders and Stroke and grant from the National Institutes of Health)	Mean age: 30.5 Mean sex: 42% male/58% female Race: 96% white EDSS Score: *3.11* Relapse rate: 3.85 over 2 years Time from diagnosis of MS: 5.5 years duration of disease Other clinical features of MS: NA	Arm 1: GA 20 mg SC daily (Copaxone) Arm 2: Placebo	Randomised 25 arm 1 25 arm 2
BRAVO 2014 RRMS (McDonald 2005)	Country: US, Bulgaria, Croatia, Czech Republic, Estonia, Georgia, Germany, Israel, Italy, Lithuania, Macedonia, Poland, Romania, Russia, Slovakia, South Africa, Spain, Ukraine and others not specified No. of countries: 18 Centres: 140 Study period: April 2008 to June 2011. 24 months follow up Sponsor: Teva Pharmaceutical Industries	Mean age: Median: 37.5 placebo, 38.5 IFN Mean sex: 71.3% females in placebo arm, 68.7% females in IFN arm Race: N/A EDSS Score: Median: 2.5 placebo, 2.5 IFN Median Relapse rate: previous year: 1.0 placebo, 1.0 IFN; previous 2 years: 2.0 placebo, 2.0 IFN Median Time from diagnosis of MS: 1.2 placebo, 1.4 IFN Other clinical features of MS: NA	Arm 1: IFN β-1a 30 μg IM once weekly (Avonex) Arm 2: Oral placebo once-daily with neurologist monitoring	Randomised 447 arm 1 450 arm 2
Calabrese 2012 RRMS (McDonald 2005)	Country: Italy No. of countries: 1 Centres: 1 Study period: 1 Jan 2007–30 June 2008 Follow up over 2 years Sponsor: grant from Merck Serono S.A	Mean age: 36.5 (9.9) Mean sex: 70.2% of female/20.8% of male Race: NA EDSS Score: 2.1 (1.1) Relapse rate: 1.2 (0.7) Time from diagnosis of MS: 5.6 years (2.4) Other clinical features of MS: None	Arm 1: IFN β-1a 44 SC three times weekly (Rebif) Arm 2: IFN β-1a 30 μg IM once weekly (Avonex) Arm 3: GA 20 mg SC daily (Copaxone)	Randomised 55 arm 1 55 arm 2 55 arm 3
CombiRx 2013 RRMS (McDonald 2001, Poser)	Country: United States, Canada No. of countries: 2 Centres: 68 Study period: January 2005–April 2012. Minimally 36 months follow up Sponsor: NIH, with materials provided by Biogen and Teva	Mean age 38.3 Mean sex: 70.3% female Race: 87.6% white EDSS Score: 2.0 Relapse rate: 1.7 relapses in last year, on average Time from diagnosis of MS: 1.2 Other clinical features of MS: NA	Arm 1: IFN β-1a 30 μg IM once weekly (Avonex) Arm 2: GA 20 mg SC daily (Copaxone)	Randomised 250 arm 1 259 arm 2
CONFIRM 2012 RRMS (McDonald 2005)	Country: USA, Belarus, Belgium, Bosnia and Herzegovina, Bulgaria, Canada, Costa Rica, Croatia, Czech Republic, Estonia, France, Germany, Greece, India, Ireland, Israel, Latvia, Macedonia, Mexico, Republic of Moldova, New Zealand, Poland, Puerto Rico, Romania, Serbia, Slovakia, Spain, Ukraine No. of countries: 28 Centres: 200 Study period: 2 year follow up Sponsor: Biogen idec	Mean age 36.8 Mean sex: 70% female Race: 84% white EDSS Score: 2.6 Relapse rate: 1.4 in prior 12 months Time from diagnosis of MS: 4.6 years Other clinical features of MS: any prior DMTs (%) = 29%	Arm 1: GA 20 mg SC daily (Copaxone) Arm 2: 2 placebo capsules orally thrice daily	Randomised 360 arm 1 363 arm 2
Cop1 MSSG 1995 RRMS (Poser)	Country: USA No. of countries: 1 Centres: 11 Study period: October, 1991, and May, 1992. 2 year follow up. Sponsor: the FDA orphan drug program, the National multiple sclerosis society, and TEVA pharmaceutical	Mean age 34.4. Mean sex: 73% female Race: 94% white EDSS Score: 2.6 Relapse rate: 2.9 prior 2-year rate MS duration:6.9 years Other clinical features of MS: ambulation index = 1.1	Arm 1: GA 20 mg SC daily (Copaxone) Arm 2: Placebo	Randomised 125 arm 1 126 arm 2
ECGASG 2001 RRMS (Poser)	Country: Canada No. of countries: 7 Centres: 29 Study period: Enrollment started in February 1997 and concluded in November 1997. 9 month follow up Sponsor: Teva Pharmaceutical Industries	Mean age 34 Mean sex: NA Race: NA EDSS Score: 2.4 Relapse rate: 2.65 Disease duration (years): 8.1 Other clinical features of MS: ambulation index = 1.15	Arm 1: GA 20 mg SC daily (Copaxone) Arm 2: Placebo SC injections	Randomised 119 arm 1 120 arm 2
Etemadifar 2006 RRMS (Poser)	Country: Iran No. of countries: 1 Centres: 1 Study period: September 2002 and September 2004. 24 month follow up Sponsor: Not specified	Mean age 28.5 Mean sex: 76% female Race: NA EDSS Score: 2.0 Relapse rate 1 year prior: 2.2 Time from diagnosis of MS: 3.2 years Other clinical features of MS: None	Arm 1: IFN β-1b 250 μg SC every other day (Betaferon) Arm 2: IFN β-1a 30 μg IM once weekly (Avonex) Arm 3: IFN β-1a 44 SC three times weekly (Rebif)	Randomised 30 arm 1 30 arm 2 30 arm 3
EVIDENCE 2007 RRMS (Poser)	Country: USA, France, UK, Norway, Austria, Germany, France, Finland, Sweden, Canada No. of countries: 10 Centres: 56 Study period: Unclear. Minimally 48 weeks follow up, average 64.2 Sponsor: Serono	Mean age 37.9 Mean sex: 74.8% female Race: 91.0% Caucasian EDSS Score: 2.3 Median: 2.0 Relapse rate: 2.6 Median 2.0 relapses in last 2 years Duration of MS: 6.6. Median: 4.0–4.1 years Other clinical features of MS: Time since last relapse (months): Median 3.9 to 4.4; mean 5.1	Arm 1: IFN β-1a 44 SC three times weekly (Rebif) Arm 2: IFN β-1a 30 μg IM once weekly (Avonex)	Randomised 339 arm 1 338 arm 2
GALA 2013 RRMS (McDonald 2005)	Country: United States, Bulgaria, Croatia, Germany, Poland, Romania, and Ukraine and others No. of countries: 17 Centres: 142 Study period: Not specified. 12 months follow up. Sponsor: TEVA pharmaceutical industries	Mean age 37.6 Mean sex: 68% female Race: 98% Caucasian EDSS Score: 2.7 Relapse rate: 1.3 in the prior 12 months, 1.9 in the prior 24 months Time from diagnosis of MS: NA Other clinical features of MS: Time from onset of first symptoms of MS = 7.7 years	Arm 1: GA 40 mg SC three times weekly (Copaxone) Arm 2: SC placebo injections	Randomised 943 arm 1 461 arm 2
GATE 2015 RRMS (McDonald 2010)	Country: USA, Belarus, Bosnia and Herzegovina, Bulgaria, Croatia, Czech Republic, Estonia, Georgia, Germany, Italy, Mexico, Republic of Moldova, Poland, Romania, Russian Federation, Serbia, South Africa, Ukraine, United Kingdom No. of countries: 20 Centres: 118 Study period: Recruited between December 7, 2011, and March 21, 2013; last follow-up December 2, 2013. Follow up 9 months (double-blind follow-up) + additional 15 months (open-label) Sponsor: Synthon BV	Mean age 33.1 Mean sex: 66.4% female Race: NA EDSS Score: 2.7 Relapse rate: 1.9 in prior 2 years Time from diagnosis of MS: NA Other clinical features of MS: • Time to onset of first symptoms to randomisation (years): 5.9 • No history of prior disease treatment: 16.1%	Arm 1: GA 20 mg SC daily (Copaxone) Arm 2: Placebo	Randomised 357 arm 1 84 arm 2
IFNB MSSG 1995 RRMS (Poser)	Country: USA and Canada No. of countries: 2 Centres: 11 Study period: after 2 years of follow-up, all subjects were given the option of continuing treatment in a double-blind fashion, extending the total treatment period to 5.5 years for some patients Sponsor: Triton Biosciences, Berlex Laboratories	Mean age 35.6 Mean sex: 70% female Race: 94% white EDSS Score: 2.9 Relapse rate: 3.5 in prior 2 years Time from diagnosis of MS:4.3 years Other clinical features of MS: Baseline Scripps neurological rating scale: 80.8	Arm 1: IFN β-1b 250 μg SC every other day (Betaferon) Arm 2: SC injections placebo	Randomised 124 arm 1 123 arm 2
IMPROVE 2012 RRMS (McDonald 2005)	Country: Italy, Germany, Serbia, Canada, Bulgaria, Estonia, Lithuania, Romania, Russia, Spain No. of countries: 10 Centres: 5 Study period: December 2006 to February 2009. Follow up 16 weeks for the double-blind phase, then 24 weeks where all patients received interferon beta 1-a, at last 4 weeks of safety period observation Sponsor: Merck Serono S.A.	Mean age NA Mean sex: NA Race: NA EDSS Score: NA Relapse rate: NA Time from diagnosis of MS: NA Other clinical features of MS: NA	Arm 1: IFN β-1a 44 SC three times weekly (Rebif) Arm 2: SC injections of placebo	Randomised 120 arm 1 60 arm 2
INCOMIN 2002 RRMS (Poser)	Country: Italy No. of countries: 1 Centres: 15 Study period: October, 1997, and June, 1999. 2 year follow up Sponsor: Istituto Superiore di Sanita’ of the Italian Ministry of Health and the Italian MS Society	Mean age 36.9 Mean sex: 65% female Race: NA EDSS Score: 1.97 Relapse rate 2 years prior: 1.45 Time from diagnosis of MS: 6.3 years Other clinical features of MS: None	Arm 1: IFN β-1b 250 μg SC every other day (Betaferon) Arm 2: IFN β-1a 30 μg IM once weekly (Avonex)	Randomised 92 arm 1 96 arm 2
Kappos 2011 RRMS (McDonald 2001)	Country: Belgium, Bulgaria, Canada, Czech Republic, Denmark, France, Germany, Italy, Mexico, Romania, Russian Federation, Serbia, Slovakia, Spain, Switzerland, Ukraine, United Kingdom, USA and others No. of countries: 20 Centres: 79 Study period: Not specified. Up to 96 weeks follow up. Sponsor: F Hoffmann-La Roche Ltd., Biogen Idec Inc	Mean age 37.5 Mean sex: 65% female Race: 96% white EDSS Score: 3.3 Relapse rate: NA Time from diagnosis of MS: median only Other clinical features of MS: NA	Arm 1: IFN β-1a 30 μg IM once weekly (Avonex) Arm 2: placebo injection every other week	Randomised 55 arm 1 54 arm 2
Knobler 1993 RRMS (Poser)	Country: USA No. of countries: 1 Centres: 3 Study period: June and October 1986. Follow up 3 years (24 weeks of initial follow-up for the 5 groups then all the patients that had received 0.8 mU, 4MU and 16MU for 24 weeks received a dose of 8MU from week 24 to 3 years) Sponsor: Triton Biosciences, Inc. and Berlex Laboratories, Inc	Mean age 35.6 Mean sex: 48% female Race: NA EDSS Score: 3.1 Mean exacerbation in prior 2 years: 2.84 Time from diagnosis of MS: 6.6 years Other clinical features of MS: mean Scripps Neurological Rating Scale (NRS): 76.6	Arm 1: IFN β-1b 250 μg SC every other day (Betaferon) Arm 2: Subcutaneous injection of placebo (1 mL like Betaseron 8 MU)	Randomised 6 arm 1 7 arm 2
MSCRG 1996 RRMS (Poser)	Country: USA No. of countries: 1 Centres: 4 Study period: November, 1990 to early 1993 2 years follow up for all-patients + 2 additional years for patients completing dosing before the end of the first period of follow-up. Sponsor: National Institutes of Health, National Institute of Neurological Disorders and Stroke (NINDS) grant R01–26321 and Biogen, Inc.	Mean age 36.8 Mean sex: 73.7% female Race: 93% white EDSS Score: *2.4* Relapse rate: 1.2 MS duration (years): 6.5 Other clinical features of MS: None	Arm 1: IFN β-1a 30 μg IM once weekly (Avonex) Arm 2: Placebo	Randomised 158 arm 1 143 arm 2
PRISMS 1998 RRMS (Poser)	Country: Australia, Belgium, Canada, Finland, Germany, Netherlands, Sweden, Switzerland, UK No. of countries: 9 Centres: 22 Study period: May 1994 to February 1995 with 2 years follow up. Sponsor: Ares- Serono	Mean age Median: 34.9 Mean sex: 69% female Race: NA EDSS Score: 2.5 (SD 1.2) Relapse rate: 3.0 (SD 1.2) Time from diagnosis of MS: Median: 5.3 years) Other clinical features of MS: NA	Arm 1: IFN β-1a 22 μg SC three times weekly (Rebif) Arm 2: IFN β-1a 44 SC three times weekly (Rebif) Arm 3: Placebo	Randomised 189 arm 1 184 arm 2 187 arm 3
REFORMS 2012 RRMS (McDonald 2005, Poser)	Country: USA No. of countries: 1 Centres: 27 Study period: December 2006–November 2007. 12 weeks follow up Sponsor: EMD Serono, Pfizer	Mean age 40.52 (SD 9.65) Mean sex: 70% female Race: 87.6% white EDSS Score: NA Relapse rate: 1.33 (SD 0.49) (of those with relapses) Time from diagnosis of MS: 1.47 yrs. (3.31) Other clinical features of MS: Percentage with no relapse in last 12 months: 24 (18.6%) Time since onset: 5.12 yrs. (6.68) Percentage diagnosed with Poser criteria: 36 (27.9%) Time since last relapse, of those with last-year relapses: 3.76 mos (2.93) Steroid treatment episodes: 0.50 (0.55) Percentage needing more than one course of steroids: 49 (38.0%)	Arm 1: IFN β-1a 44 SC three times weekly (Rebif) Arm 2: IFN β-1b 250 μg SC every other day (Betaferon)	Randomised 65 arm 1 64 arm 2
REGARD 2008 RRMS (McDonald 2001)	Country: Argentina, Austria, Brazil, Canada, France, Germany, Ireland, Italy, Netherlands, Russia, Spain, Switzerland, UK, and USA No. of countries: 14 Centres: 80 Study period: February and December 2004, with 96 weeks follow up Sponsor: EMD Serono, Pfizer	Mean age 36.8 Mean sex: 29.5% male Race: 93.6% white EDSS Score: 2.34 Relapse rate: Presented as distribution of relapses; months since last relapse about 5 on average Time from diagnosis of MS: Years since first relapse: 6.2 Other clinical features of MS: Receiving steroid treatment in last 6 months: 43.7%	Arm 1: IFN β-1a 44 SC three times weekly (Rebif) Arm 2: GA 20 mg SC daily (Copaxone)	Randomised 386 arm 1 378 arm 2

*RRMS* relapsing remitting MS, *SPMS* secondary progressive MS, *CIS* clinically isolated syndrome, *IFN* interferon, *GA* glatiramer acetate, *IM* intramuscular, *SC* subcutaneous, *NA* not available, *EDSS* Expanded Disability Status Score

### Risk of bias

Risk of bias assessments are detailed in Table 2. All studies that adequately detailed their method of randomisation (*n* = 15, 63%) were appraised as being at low risk of bias in this domain. A similar number of studies (*n* = 15) were judged to be at low risk of bias from allocation concealment, though one study (Bornstein 1987 [18]) was classed as at high risk of bias in this domain. We judged that most studies were at high risk of bias in blinding of participants and personnel (*n* = 24, 83%) and blinding of outcome assessment (*n* = 18, 75%) due to a combination of injection site reactions in placebo-controlled trials and an open label design. Five studies (21%) were at high risk of bias from incomplete outcome data due to differential attrition between arms, and we believed that four studies (17%) were at high risk of bias from selective reporting. Finally, most studies (*n* = 17, 71%) were at high risk of bias from other sources, generally stemming from industry sponsorship.

**Table 2:** Risk of bias judgments for included studies

Reference	Random sequence generation	Allocation concealment	Blinding of participants and personnel	Blinding of outcome assessment	Incomplete outcome data	Selective reporting	Other sources of bias
ADVANCE 2014	Low risk	Low risk	High risk	High risk	High risk	Low risk	High risk
AVANTAGE 2014	Unclear risk	Unclear risk	High risk	High risk	Unclear risk	Low risk	Low risk
BECOME 2009	Unclear risk	Unclear risk	High risk	High risk	Low risk	Low risk	High risk
BEYOND 2009	Low risk	Low risk	High risk	High risk	Unclear risk	Low risk	High risk
Bornstein 1987	Unclear risk	High risk	High risk	High risk	Low risk	Low risk	Low risk
BRAVO 2014	Low risk	Low risk	High risk	Low risk	Low risk	High risk	High risk
Calabrese 2012	Low risk	Low risk	High risk	High risk	Low risk	High risk	Low risk
CombiRx 2013	Low risk	Low risk	Low risk	Low risk	High risk	Low risk	Low risk
CONFIRM 2012	Low risk	Low risk	High risk	High risk	High risk	Low risk	High risk
Cop1 MSSG 1995	Unclear risk	Low risk	High risk	High risk	Low risk	Low risk	Low risk
ECGASG 2001	Low risk	Low risk	High risk	High risk	Low risk	Unclear risk	High risk
Etemadifar 2006	Unclear risk	Unclear risk	High risk	High risk	Low risk	Unclear risk	Unclear risk
EVIDENCE 2007	Low risk	Low risk	High risk	High risk	Low risk	Low risk	High risk
GALA 2013	Unclear risk	Low risk	High risk	High risk	Unclear risk	Low risk	High risk
GATE 2015	Low risk	Low risk	High risk	High risk	Low risk	Low risk	High risk
IFNB MSSG 1995	Unclear risk	Unclear risk	High risk	High risk	High risk	Low risk	High risk
IMPROVE 2012	Unclear risk	Low risk	Unclear risk	Unclear risk	Unclear risk	High risk	High risk
INCOMIN 2002	Low risk	Low risk	High risk	High risk	Low risk	Low risk	Low risk
Kappos 2011	Low risk	Low risk	High risk	High risk	Low risk	High risk	High risk
Knobler 1993	Unclear risk	Unclear risk	High risk	High risk	Unclear risk	Unclear risk	High risk
MSCRG 1996	Low risk	Unclear risk	Low risk	Low risk	High risk	Unclear risk	High risk
PRISMS 1998	Low risk	Low risk	Unclear risk	Low risk	Low risk	Low risk	High risk
REFORMS 2012	Low risk	Unclear risk	High risk	High risk	Unclear risk	Low risk	High risk
REGARD 2008	Low risk	Unclear risk	High risk	Low risk	Low risk	Low risk	High risk

### Annualised relapse rates

**Fig. 2:**
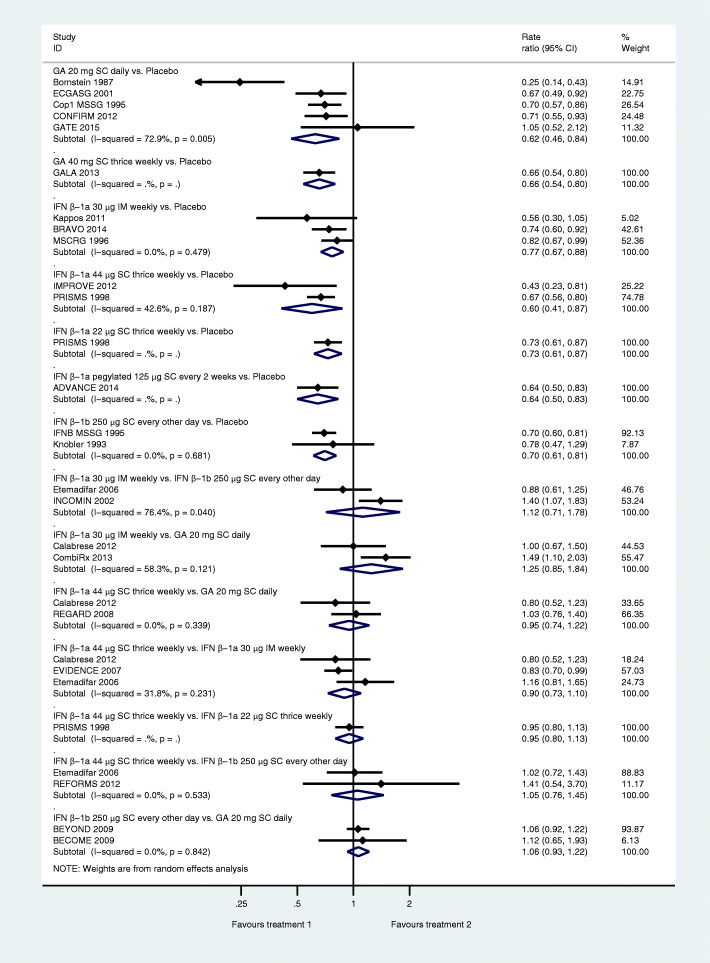
Pairwise meta-analyses for annualised relapse rate. IFN: interferon, GA: glatiramer acetate, IM: intramuscular, SC: subcutaneous

Direct evidence from comparisons is shown in Fig. 2. All drugs had a beneficial effect on ARR as compared to placebo. None of the pooled comparisons showed evidence of a statistically significant effect favouring one drug over another drug. Heterogeneity quantified by I^2^ ranged from 0% (IFN β-1b 250 μg SC every other day, IFN β-1a 30 μg IM once a week) to 43% (IFN β-1a 44 μg SC thrice weekly) and 73% (GA 20 mg SC once daily). However, there were too few studies in each comparison to enable exploration of heterogeneity.

Findings derived from the NMA for comparisons between each drug and placebo substantially mirrored those of the pairwise comparisons, and reflected statistically significant reductions in ARR in patients receiving active drugs (see Table 3). There was little evidence of superiority of one drug over another. However, GA 20 mg SC once daily (RR = 0.82, 95% CI [0.73, 0.93]), IFN β-1a 44 μg SC thrice weekly (0.85, [0.76, 0.95]) and IFN β-1b 250 μg SC every other day (0.86, [0.76, 0.97]) all produced significant reductions in ARR as compared to IFN β-1a 30 μg IM once a week. Ranking of the drugs suggested that the drug with the highest cumulative probability of superiority was GA 20 mg SC once daily. We found no evidence of inconsistency.

**Table 3:** Network meta-analysis results for annualised relapse rate^a^

Drug	SUCRA	GA 20 mg daily	PegIFN β-1a 125 μg every 2 weeks	GA 40 mg thrice weekly	IFN β-1a 44 μg SC thrice weekly	IFN β-1b 250 μg SC every other day	IFN β-1a 22 μg SC thrice weekly	IFN β-1a 30 μg IM weekly	Placebo
GA 20 mg daily	0.77		1.01 (0.77, 1.33)	1.00 (0.80, 1.24)	0.97 (0.85, 1.10)	0.95 (0.86, 1.05)	0.91 (0.76, 1.08)	0.82 (0.73, 0.92)	0.65 (0.59, 0.72)
PegIFN β-1a 125 μg every 2 weeks	0.73			0.98 (0.71, 1.35)	0.95 (0.72, 1.26)	0.94 (0.71, 1.23)	0.89 (0.66, 1.21)	0.81 (0.62, 1.06)	0.64 (0.50, 0.83)
GA 40 mg thrice weekly	0.70				0.97 (0.77, 1.22)	0.96 (0.77, 1.19)	0.91 (0.71, 1.17)	0.82 (0.66, 1.03)	0.66 (0.54, 0.80)
IFN β-1a 44 μg SC thrice weekly	0.64					0.99 (0.86, 1.13)	0.94 (0.80, 1.10)	0.85 (0.76, 0.95)	0.68 (0.60, 0.76)
IFN β-1b 250 μg SC every other day	0.56						0.95 (0.79, 1.14)	0.86 (0.76, 0.97)	0.69 (0.62, 0.76)
IFN β-1a 22 μg SC thrice weekly	0.43							0.91 (0.76, 1.08)	0.72 (0.61, 0.85)
IFN β-1a 30 μg IM weekly	0.18								0.80 (0.72, 0.88)
Placebo	0								
Test for inconsistency (χ^2^, df, p)		11.71, 11, 0.38							

^a^Findings are expressed as rate ratio (RR) with 95% CI

*IFN* interferon, *GA* glatiramer acetate, *IM* intramuscular, *SC* subcutaneous, *SUCRA* surface under the cumulative ranking curve

### Sensitivity analyses

Several characteristics of the trials included in this network suggested that additional analyses would confirm the robustness of our findings. All of these analyses were post hoc. First, after exclusion of the REFORMS 2012 [19] trial from the analysis (where relapses were self-reported by subjects instead of being documented by an examining neurologist), effect estimates remained essentially unchanged for all pairwise comparisons. Second, we compared findings for studies with ‘true’, blinded placebos against studies that did not have blinded placebos. That is, several studies did not deliver placebos via the same route of administration [14–16]. We found that effects for these drugs against placebo were robust to inclusion of a covariate in the model for trials without a blinded placebo. Third, after exclusion of the Bornstein 1987 [18] trial that was an outlier in the comparison between GA 20 mg SC once daily and placebo, the pooled rate ratio for relapses still suggested a reduction in ARR as compared to placebo (RR = 0.71, 95% CI [0.62, 0.82]), with I^2^ of 0% (see Additional file 2). Re-estimation of the NMA yielded a change in the SUCRA-based rankings, with GA 20 mg SC once daily now ranked third, but point estimates and confidence intervals were not substantially different in the new model.

### Time to progression confirmed at three months

**Fig. 3:**
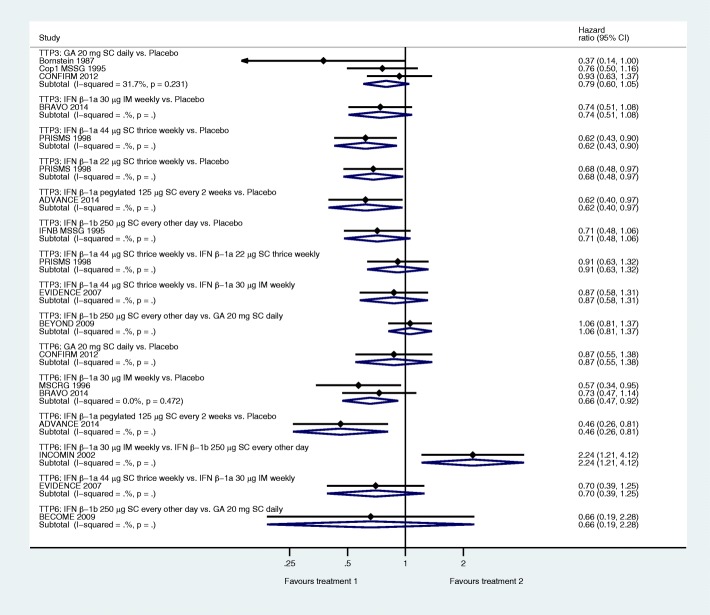
Pairwise meta-analyses for time to progression. IFN: interferon, GA: glatiramer acetate, IM: intramuscular, SC: subcutaneous; TTP3: time to progression confirmed at 3 months; TTP6: time to progression confirmed at 6 months

Direct evidence from comparisons is shown in Fig. 3. GA 40 mg thrice weekly was not represented in this analysis. Comparison of drugs against placebo showed a mixed pattern of results. None of the three direct comparisons between active drugs suggested a benefit of one over another. Most comparisons were informed by only one study.

Comparisons for active drugs vs. placebo were similar between the NMA and the pairwise meta-analyses (see Table 4). Notably, additional information from indirect comparisons yielded a more precise estimate of effectiveness for both IFN β-1a 30 μg IM once a week vs placebo (HR = 0.73, 95% CI [0.53, 1.00], *p* = 0.0499) and GA 20 mg SC once daily (0.76, [0.60, 0.97]). Comparisons between active drugs estimated from the NMA did not indicate that any one drug was statistically better than the others, but ranking of the drugs suggested that the drug with the highest cumulative probability of superiority was IFN β-1a 44 μg SC thrice weekly. We found no evidence of inconsistency.

**Table 4:** Network meta-analysis results for time to progression^a^

Time to progression confirmed at 3 months									
Drug	SUCRA	IFN β-1a 44 μg SC thrice weekly	PegIFN β-1a 125 μg every 2 weeks	IFN β-1a 22 μg SC thrice weekly	IFN β-1a 30 μg IM weekly	GA 20 mg daily	IFN β-1b 250 μg SC every other day	Placebo	GA 40 mg SC thrice weekly
IFN β-1a 44 μg SC thrice weekly	0.77		1.01 (0.59, 1.74)	0.92 (0.65, 1.30)	0.86 (0.62, 1.19)	0.82 (0.56, 1.22)	0.81 (0.53, 1.22)	0.63 (0.46, 0.86)	Not included in this analysis
PegIFN β-1a 125 μg every 2 weeks	0.75			0.91 (0.52, 1.59)	0.85 (0.49, 1.46)	0.81 (0.49, 1.34)	0.80 (0.47, 1.34)	0.62 (0.40, 0.97)	
IFN β-1a 22 μg SC thrice weekly	0.62				0.94 (0.62, 1.42)	0.90 (0.59, 1.36)	0.88 (0.57, 1.36)	0.68 (0.49, 0.96)	
IFN β-1a 30 μg IM weekly	0.5					0.96 (0.65, 1.42)	0.94 (0.62, 1.43)	0.73 (0.53, 1.00)*	
GA 20 mg daily	0.44						0.98 (0.78, 1.24)	0.76 (0.60, 0.97)	
IFN β-1b 250 μg SC every other day	0.39							0.78 (0.59, 1.02)	
Placebo	0.02								
Test for inconsistency (χ2, df, p)		0.35, 2, 0.84							
Time to progression confirmed at 6 months									
Drug	SUCRA	IFN β-1b 250 μg SC every other day	PegIFN β-1a 125 μg every 2 weeks	IFN β-1a 44 μg SC thrice weekly	IFN β-1a 30 μg IM weekly	GA 20 mg daily	Placebo	PegIFN β-1a 125 μg every 2 weeks	GA 40 mg thrice weekly
IFN β-1b 250 μg SC every other day	0.9		0.74 (0.32, 1.71)	0.71 (0.32, 1.60)	0.50 (0.29, 0.87)	0.42 (0.21, 0.83)	0.34 (0.18, 0.63)	Not included in this analysis	
PegIFN β-1a 125 μg every 2 weeks	0.71			0.97 (0.40, 2.33)	0.68 (0.35, 1.31)	0.56 (0.28, 1.15)	0.46 (0.26, 0.81)		
IFN β-1a 44 μg SC thrice weekly	0.7				0.70 (0.39, 1.25)	0.58 (0.27, 1.27)	0.47 (0.24, 0.93)		
IFN β-1a 30 μg IM weekly	0.4					0.83 (0.49, 1.41)	0.68 (0.49, 0.94)		
GA 20 mg daily	0.25						0.82 (0.53, 1.26)		
Placebo	0.05								
Test for inconsistency (χ2, df, p)		0.77, 1, 0.38							

^a^Findings are presented as HR (95% CI)

*IFN* interferon, *GA* glatiramer acetate, *IM* intramuscular, *SC* subcutaneous, *SUCRA* surface under the cumulative ranking curve

### Time to progression confirmed at six months

Direct evidence from comparisons is shown in Fig. 3. All comparisons drew from a single study, except for IFN β-1a 30 μg IM once a week as compared to placebo. Only three drugs, GA 20 mg SC one daily, IFN β-1a 30 μg SC once weekly and IFN β-1a pegylated 125 μg every 2 weeks, were compared against placebo.

In the NMA, estimates for GA 20 mg SC once daily (HR = 0.82, 95% CI [0.53, 1.26]), IFN β-1a 30 μg IM once a week (0.68, [0.49, 0.94]) and IFN β-1a pegylated 125 μg every 2 weeks (0.46, [0.26, 0.81]) compared to placebo mirrored the direct evidence (see Table 4). Indirect comparisons suggested that both IFN β-1a 44 μg SC thrice weekly (0.47, [0.24, 0.93]) and IFN β-1b 250 μg SC every other day (0.34, [0.18, 0.63]) showed evidence of delaying disability progression as compared to placebo. The NMA suggested that IFN β-1b 250 μg SC every other day was superior both to IFN β-1a 30 μg IM once a week (HR = 0.50, 95% CI [0.29, 0.87]) and to GA 20 mg SC once daily (0.41, [0.21, 0.83]), but these findings were driven by the INCOMIN 2002 trial [20] and relied on a hazard ratio estimated from summary statistics. Ranking of the drugs suggested that the drug with the highest cumulative probability of superiority was IFN β-1b 250 μg SC every other day. Tests of inconsistency in the network did not suggest that direct and indirect evidence were in disagreement; however, the network was sparse and only one comparison included more than one study.

### Discontinuation due to AEs

Two NMA models were estimated: one for studies with 24-month follow-up and one including all studies with the follow-up of greatest maturity. Neither NMA found evidence that one drug was more likely to lead to discontinuation than another. However, confidence intervals were wide and NMA-based estimates were often numerically different to estimates from the direct evidence alone. Moreover, both networks of evidence included some indication of inconsistency. In the 24-month follow-up model, the sidesplitting test suggested that direct and indirect evidence were in conflict for the comparison between GA 20 mg SC once daily and placebo, with indirect evidence suggesting that risk of discontinuation due to AEs was higher than presented in the direct evidence (*p* = 0.037). In the all-studies model, the overall Wald test suggested some signal of inconsistency (*p* = 0.09), though sidesplitting tests did not indicate an obvious source of inconsistency. Full results are in Additional file 2.

## Discussion

Meta-analyses confirmed that the different formulations of IFN-β and GA reduce ARR and generally delay progression as defined in these trials. There was little evidence that any one drug was superior to others, except for progression confirmed at 6 months, but networks were especially sparse. Findings for discontinuations due to AEs, which are intended to be indicative, did not suggest that one drug was more likely to result in discontinuation than another, but these findings relied on networks with some limited evidence of inconsistency.

### Challenges with the clinical evidence

These conclusions are tempered by several considerations. Analyses did not show a clear ‘winner’ across outcomes, and, again, comparisons between drugs estimated as part of NMA models were in the main inconclusive. Though the main model for ARR was relatively well populated, analyses for time to progression confirmed at six months were especially sparse. In particular, several comparisons of drugs vs. placebo estimated as part of this last model relied exclusively on indirect evidence. Moreover, analyses for time to progression confirmed at three and at six months did not show a consistent pattern, except that all drugs were beneficial in delaying progression where progression was defined using the EDSS. This is particularly concerning, as progression confirmed at six months is considered to be a ‘stronger’ outcome than progression confirmed at three months.

Measurement of disease progression also relied on the EDSS, a measure that, while broadly accepted in clinical trials, may be of dubious value in measuring disability per se. The EDSS is heavily weighted towards mobility over other important aspects of disability affected by disease progression in MS, such as cognitive function. Additionally, progression outcomes based on confirmed progression at 3 or 6 months overestimate the accumulation of permanent disability by up to 30% [21]. This is in part because recovery from relapses may take longer than several months, and thus ‘confirmed’ progression may reflect residual relapse-related symptoms. Consequently, while time to progression confirmed at 3 or 6 months may be standard within the relatively short timeframe of clinical trials, these outcomes may not capture the true accumulation of MS-related disability over the lifecourse, and thus true differences between DMTs in delaying disease progression.

NMA models also had imbalanced risk of bias across the networks of studies. For example, most trials comparing two active treatments were open-label, whereas most trials comparing active treatments against placebos were blinded. Many trials relied on short follow-up, generally less than two years in duration, which increases the risk of spurious results [21]. Thus, participants were aware of the drugs they were receiving. This might have posed a greater risk for unblinding of outcome assessors than in ostensibly double-blinded trials. In addition, the majority of studies were judged as high risk of bias under the ‘other’ category of the Cochrane tool given that most of these were funded by drug companies. Although no research has specifically been undertaken in the field of MS trials, empirical examination of trials suggests that industry-sponsored RCTs are more likely to have favourable results than non-industry sponsored RCTs [2]. A final issue is that patient populations recruited into trials may not be the same over time, given the nearly 20-year span of the trials included in our models. These differences may well extend to diagnostic definitions of MS, and detection and diagnosis of relapses and disease progression. Again, insufficient studies on each pairwise comparison prevented exploration of this problem, but it is conceivable that this might have affected transitivity of our networks of evidence.

### Review-level strengths and limitations

We used a rigorous and exhaustive search to locate primary studies, which included updating existing high-quality systematic reviews. Additionally we used auditable and transparent methods to include and synthesise studies. Where appropriate, we undertook post hoc sensitivity analyses in our clinical effectiveness assessments to check the robustness of our findings. However, a limitation of our work, inherent to all systematic reviews, is publication bias. Methods for detecting publication bias in NMAs are still in development, and we did not have enough studies in any one comparison to test for small-study bias. This may be especially relevant since many of the early trials of IFN and GA for MS were small trials. Another important limitation was the selective and inconsistent reporting of outcomes. For example, one of the reasons we did not undertake a meta-analysis of time to first relapse is that there was inconsistent and often poor reporting, especially across multiple reports of the same study, which prevented imputation of hazard ratios. We were also unable to obtain meta-analysable data for one study [12], due to the tight timeline within which the original work was undertaken.

Our analysis methods had a number of statistical advantages as well as some limitations. In examining the effect of IFN and GA on progression, we used time to event outcomes and hazard ratios instead of calculating risk ratios or odds ratios at different follow-up points. Thus, trial findings were reported at their fullest ‘maturity’ [22] and all relevant data were included. We were unable to verify empirically whether hazard ratios and rate ratios were time-varying due to few comparisons on every node of the study networks. On the other hand, we judged that stratifying analyses by time to follow-up would have resulted in excessively sparse networks that would have been difficult to interpret collectively. Thus, our decision to pool study estimates across follow-up times for analyses of clinical outcomes was both a strength and a potential limitation. Notably, we stratified analyses by time to follow-up in NMAs of discontinuations due to AEs, because we judged that the only feasible estimator in these analyses was the risk ratio.

### Deviations from protocol

In our protocol, we specified that the comparator of interest was best supportive care without DMTs. In practice, this includes both best supportive care and also placebo, as reported in included trials. Though we sought to examine 10 outcomes relevant to RRMS in our original protocol, we report here findings for relapse rate, disability and discontinuation due to adverse events, as synthesis for other outcomes was limited and in many parts meta-analysable. Detailed findings for each of these outcomes are available in the main report [2]. Moreover, disability was ultimately measured and included in these meta-analyses as ‘time to progression’, as this was the most common outcome across trials. Finally, we implemented network meta-analyses in a frequentist paradigm rather than using WinBUGS as specified in the protocol.

### In relation to research and practice

Our findings updated prior reviews, though comparability of findings is limited. We included trials examining IFN and GA against each other and against a no-treatment comparator, and restricted inclusion to doses and formulations within their marketing authorisation as compared to Tramacere et al. [3] who broadly examined immunomodulators and immunosuppressants for RRMS. Because they included studies across drugs and because they used risk ratios as the sole outcome estimator, our analyses and theirs are largely incommensurate. Our systematic review and NMA may however offer more clinically relevant evidence because of our focus on doses used in clinical practice. However, our analyses for discontinuation due to AEs agreed with theirs. Neither review suggested that any one drug had a significant effect on discontinuation due to AEs relative to placebo.

Our findings agree with the ABN guidelines [1] in that the guidelines classify IFN-β and GA as drugs of ‘moderate efficacy’, and observe that there is not much data to support differences in effectiveness between them. Our analysis does suggest that these drugs are effective in reducing relapse rate, which may have an effect on progression.

Longer-term observational cohorts have also examined DMT effectiveness over time and shed some doubt on the findings from randomised trials. In the year 8 analyses from the UK Risk Sharing Scheme, DMTs were not found to be cost-effective and the drugs assessed were not substantially different in terms of delays in disease progression (personal communication with UK Department of Health, 2016). An analysis from the MSBase study, an international registry with ‘real-world’ data from MS patients, has suggested that GA or subcutaneous IFN-β-1a are more effective in controlling relapse rate than other IFN-β, though drugs were not different on disease progression [23]. While this analysis relied on matching to overcome lack of randomisation, a strength is that it used disability progression confirmed at 12 months instead of at 3 or 6 months.

### Future research

First, findings from this review will require updating as generic versions of the DMTs considered here are authorised. For example, the GATE trial also tested a generic version of glatiramer acetate against the branded version and placebo [24]. Key flaws in the assembled clinical effectiveness evidence included the lack of long-term follow-up and the absence of a measure for disease progression adequately capturing worsening of disability. A large-scale, longitudinal randomised trial comparing active first-line agents and using clinically meaningful and robust measures of disability progression would contribute towards resolving uncertainty about the relative benefits of different IFN or GA formulations (and other first line agents). While other, newer first line agents were beyond the remit of our systematic review, few randomised comparisons exist and thus a large trial could resolve remaining questions of comparative effectiveness. It may also be that using standardised definitions for relapses and disease progression together with blinded adjudicator panels could attenuate the risk of bias accruing to an open-label trial. Because of this lack of long-term follow-up, DMT trials are not informative on whether drugs delay progression to SPMS. Understanding long-term effectiveness of DMTs as described above would will also provide better information for informing cost-effectiveness evaluations, the effectiveness estimates for which currently rely on extrapolation from short-term trials. Use of a more relevant measure for disability and disease progression, especially as regards the development of secondary progressive MS, will also lead to better and more robust valuation of benefits accruing from DMTs.

Finally, above and beyond the broad interpretation that DMTs reduce ARR, there is a need to understand who responds best to DMTs; especially who does not respond to IFN or GA early on, to enable more targeted therapeutic decisions. Though several trials included in our clinical effectiveness review used subgroup analyses, based for example, on presenting lesions or demographic characteristics, a more fine-grained understanding can help patients and clinicians make better-informed decisions.

## Conclusions

Our meta-analyses confirmed that IFN-β and GA reduce ARR and generally delay progression as defined in these trials. We found, however, that there was no clear ‘winner’ across outcomes, and our findings were qualified by the high risk of bias across studies, and the use of an impairment/mobility scale to measure disease progression. Future research should consider more relevant measures of disability and, given that most trials have been short-term, consider a longitudinal approach to comparative effectiveness.

## Additional files

Additional file 1: Detailed search and data preparation methods. This file includes search strings, grey literature search sources, a sample data extraction form, and additional details on the statistical procedures undertaken to prepare study data for meta-analysis. (DOCX 47 kb)

Additional file 2: Additional results. This file includes detailed reasons for exclusion, tables of included publications, and sensitivity analyses for ARR, and detailed findings for discontinuation due to adverse events. (DOCX 265 kb)

## Abbreviations

ABN: Association of British Neurologists
AEs: Adverse events
ARR: Annualised relapse rate
DMT: Disease-modifying therapy
EDSS: Expanded Disability Status Scale
EMA: European Medicines Agency
GA: Glatiramer acetate
HR: Hazard ratio
IFN-β: Beta-interferon
NMA: Network meta-analysis
RR: Rate ratio
RRMS: Relapsing-remitting multiple sclerosis
SPMS: Secondary progressive multiple sclerosis
SUCRA: Surface under the cumulative ranking curve
