# Supplementary material for: Comparative effectiveness of beta-interferons and glatiramer acetate for relapsing-remitting multiple sclerosis: systematic review and network meta-analysis of trials including recommended dosages
Source: BMC Neurol. 2018 Oct 3;18:162. doi: 10.1186/s12883-018-1162-9 (PMC6169084; doi:10.1186/s12883-018-1162-9)
Supplement: Supplementary file 1 — Detailed search and data preparation methods. This file includes search strings, grey literature search sources, a sample data extraction form, and additional details on the statistical procedures undertaken to prepare study data for meta-analysis. (DOCX 47 kb) [file 12883_2018_1162_MOESM1_ESM.docx]

**Additional file 1: detailed search and data preparation methods**

Appendix S1. Search strategy

Appendix S2. Data extraction

Appendix S3. Data preparation

**Appendix S1. Search strategies**

We ran electronic searches on the following databases:

- Cochrane Multiple Sclerosis and Rare Diseases of the CNS group specialized register
- MEDLINE (Ovid)
- MEDLINE In-Process & Other Non-Indexed Citations (Ovid)
- Embase (Ovid)
- Cochrane Library (Wiley), including Cochrane Database of Systematic Reviews, CENTRAL, DARE, NHS EED, and HTA databases
- Science Citation Index and Conference Proceedings - Science (Web of Science)
- UKCRN Portfolio Database

We also searched the trial registers at ClinicalTrials.gov and WHO ICTRP.

Medline (Ovid), searched 27/01/2016

Exact database: Ovid MEDLINE(R) 1946 to January Week 2 2016

| 1 | exp Multiple Sclerosis/ | 46764 |
| --- | --- | --- |
| 2 | multiple sclerosis.tw. | 49799 |
| 3 | 1 or 2 | 57188 |
| 4 | randomized controlled trial.pt. | 403450 |
| 5 | controlled clinical trial.pt. | 89937 |
| 6 | clinical trial, phase ii/ or clinical trial, phase iii/ or clinical trial, phase iv/ | 35683 |
| 7 | (random* or "controlled trial*" or "clinical trial*" or rct).tw. | 873696 |
| 8 | 4 or 5 or 6 or 7 | 1065585 |
| 9 | Animals/ | 5743229 |
| 10 | Humans/ | 15593111 |
| 11 | 9 not 10 | 4140900 |
| 12 | 8 not 11 | 964542 |
| 13 | 3 and 12 | 4921 |
| 14 | (metaanalys* or "meta analys*" or "meta-analys*").tw. | 69140 |
| 15 | "systematic* review*".mp. | 61461 |
| 16 | meta analysis.pt. | 60117 |
| 17 | 14 or 15 or 16 | 122687 |
| 18 | 3 and 17 | 635 |
| 19 | limit 3 to systematic reviews | 1136 |
| 20 | 18 or 19 | 1233 |
| 21 | 13 or 20 | 5694 |
| 22 | limit 21 to yr="2012 -Current" | 1545 |

Medline In-Process & Other Non-Indexed Citations (Ovid), searched 27/01/2016

Exact database: Ovid MEDLINE(R) In-Process & Other Non-Indexed Citations January 26, 2016

| 1 | multiple sclerosis.tw. | 4892 |
| --- | --- | --- |
| 2 | (random* or "controlled trial*" or "clinical trial*" or rct).tw. | 108317 |
| 3 | 1 and 2 b | 610 |
| 4 | (metaanalys* or "meta analys*" or "meta-analys*").tw. | 14094 |
| 5 | "systematic* review*".tw. | 15189 |
| 6 | 4 or 5 | 23570 |
| 7 | 1 and 6 | 118 |
| 8 | 3 or 7 | 684 |
| 9 | limit 8 to yr="2012 -Current" | 563 |

Embase (Ovid), searched 27/01/2016

Exact database: Embase 1974 to 2016 Week 04

| 1 | *multiple sclerosis/ | 64389 |
| --- | --- | --- |
| 2 | multiple sclerosis.tw. | 80240 |
| 3 | 1 or 2 | 87466 |
| 4 | randomized controlled trial/ | 392971 |
| 5 | (random* or "controlled trial*" or "clinical trial*" or rct).tw. | 1306964 |
| 6 | 4 or 5 | 1388801 |
| 7 | 3 and 6 | 8813 |
| 8 | meta analysis/ | 103317 |
| 9 | (metaanalys* or "meta analys*" or "meta-analys*").tw. | 110582 |
| 10 | "systematic review"/ | 100520 |
| 11 | "systematic* review*".tw. | 96391 |
| 12 | 8 or 9 or 10 or 11 | 222654 |
| 13 | 3 and 12 | 1280 |
| 14 | 7 or 13 | 9616 |
| 15 | limit 14 to yr="2012 -Current" | 4527 |
| 16 | limit 15 to (conference abstract or conference paper or conference proceeding) | 2363 |
| 17 | 15 not 16 | 2164 |

Cochrane Library (Wiley), searched 27/01/2016

| ID | Search | Hits |
| --- | --- | --- |
| #1 | MeSH descriptor: [Multiple Sclerosis] explode all trees | 1916 |
| #2 | multiple sclerosis:ti,ab,kw (Word variations have been searched) | 4921 |
| #3 | #1 or #2 | 4925 |
| #4 | #1 or #2 Publication Year from 2012 to 2016 | 1861 |

Distribution of results from Cochrane Library search:

- Cochrane Reviews (44)
  - Reviews (39)
  - Protocols (5)
- Other Reviews (DARE) (60)
- Trials (CENTRAL) (1702)
- Methods Studies (0)
- Technology Assessments (HTA Database) (28)
- Economic Evaluations (27)
- Cochrane Groups (0)

Science Citation Index (Web of Knowledge), searched 27/01/2016

| # 11 | 3,248 | #9 not #10  Indexes=SCI-EXPANDED Timespan=All years |
| --- | --- | --- |
| # 10 | 237 | (#9) *AND* DOCUMENT TYPES: (Meeting Abstract OR Proceedings Paper)  *Indexes=SCI-EXPANDED Timespan=All years* |
| # 9 | [3,](http://apps.webofknowledge.com/summary.do?product=WOS&doc=1&qid=21&SID=Z1qLPVRnuodvzR2ooLE&search_mode=AdvancedSearch&update_back2search_link_param=yes)485 | #8  *Indexes=SCI-EXPANDED Timespan=2012-2016* |
| # 8 | [9,](http://apps.webofknowledge.com/summary.do?product=WOS&doc=1&qid=20&SID=Z1qLPVRnuodvzR2ooLE&search_mode=CombineSearches&update_back2search_link_param=yes)263 | #7 OR #6  *Indexes=SCI-EXPANDED Timespan=All years* |
| # 7 | [1,](http://apps.webofknowledge.com/summary.do?product=WOS&doc=1&qid=19&SID=Z1qLPVRnuodvzR2ooLE&search_mode=CombineSearches&update_back2search_link_param=yes)326 | #5 AND #1  *Indexes=SCI-EXPANDED Timespan=All years* |
| # 6 | [8,425](http://apps.webofknowledge.com/summary.do?product=WOS&doc=1&qid=9&SID=Z1qLPVRnuodvzR2ooLE&search_mode=CombineSearches&update_back2search_link_param=yes) | #2 AND #1  *Indexes=SCI-EXPANDED Timespan=All years* |
| # 5 | [216,848](http://apps.webofknowledge.com/summary.do?product=WOS&doc=1&qid=18&SID=Z1qLPVRnuodvzR2ooLE&search_mode=AdvancedSearch&update_back2search_link_param=yes) | #4 OR #3  *Indexes=SCI-EXPANDED Timespan=All years* |
| # 4 | [166,410](http://apps.webofknowledge.com/summary.do?product=WOS&doc=1&qid=6&SID=Z1qLPVRnuodvzR2ooLE&search_mode=AdvancedSearch&update_back2search_link_param=yes) | TS=(metaanalys* or meta-analys* or (meta NEAR/1 analys*))  *Indexes=SCI-EXPANDED Timespan=All years* |
| # 3 | 80,440 | TS=(systematic* NEAR/1 review*)  *Indexes=SCI-EXPANDED Timespan=All years* |
| # 2 | [1,388,789](http://apps.webofknowledge.com/summary.do?product=WOS&doc=1&qid=2&SID=Z1qLPVRnuodvzR2ooLE&search_mode=AdvancedSearch&update_back2search_link_param=yes) | TS=(random* or (clinical NEAR/1 trial*) or (controlled NEAR/1 trial*) or rct)  *Indexes=SCI-EXPANDED Timespan=All years* |
| # 1 | [85,913](http://apps.webofknowledge.com/summary.do?product=WOS&doc=1&qid=1&SID=Z1qLPVRnuodvzR2ooLE&search_mode=AdvancedSearch&update_back2search_link_param=yes) | TS="multiple sclerosis"  *Indexes=SCI-EXPANDED Timespan=All years* |

UKCRN, searched 27/01/2016

Search:

Keyword: multiple sclerosis

AND

Status: closed

AND

Study Design: Interventional

Total: 41

Cochrane MS group register of trials, searched 26/02/2016

Keywords

(interferon\*) OR (interferon beta) OR (beta-1 interferon) OR (beta 1 interferon) OR (interferon beta-1\*) OR (rebif) OR (avonex) OR (Betaseron) OR (beta-seron) OR (betaferon) OR (beta-IFN-1\*) OR (interferon beta-1\*) OR (Interferon-beta\*) OR (interferon beta\*) OR (recombinant interferon beta-1\*) OR (beta-1a interferon) OR (beta 1a interferon) OR (interferon beta-1a) OR (beta 1b interferon) OR (interferon beta1b ) OR (IFNb-1b) OR (IFNbeta-1b) OR (interferon beta-1b) OR (copolymer-1) OR (cop-1) OR (copaxone) OR (glatiramer acetate) OR (cpx) OR (cop1) OR (copolymer) OR (glatiramer) OR (polyethylene glycol-interferon-beta-1a) OR (PEG IFN-beta-1a) OR (Pegylated interferon beta-1a) OR (Ocrelizumab)

AND

(relapsing remitting) OR (relapsing-remitting ) OR (remitting-relapsing) OR (remitting relapsing) OR (secondary progressive)

Total: 265

ClinicalTrials.gov, searched 03/05/2016

Advanced Search

182 studies found for: Interventional Studies | multiple sclerosis OR clinically isolated syndrome OR CNS demyelinating OR transverse myelitis OR neuromyelitis optica | interferon OR glatiramer OR betaferon OR betaseron OR avonex OR plegridy OR rebif OR extavia OR copaxone | Phase 2, 3, 4

**WHO ICTRP, searched 14/07/2016**

(Relapsing Remitting Multiple Sclerosis OR RRMS OR clinically isolated syndrome OR CNS demyelinating OR transverse myelitis OR neuromyelitis optica) in the Condition

AND

(interferon OR glatiramer OR betaferon OR betaseron OR avonex OR plegridy OR rebif OR extavia OR copaxone) in the Intervention

588 records for 175 trials found

Websites searched

|  | **Name (Brand)** | **Website address** | **Date searched** |
| --- | --- | --- | --- |
| **Companies sponsors** | Bayer (BETAFERON) | http://www.bayer.co.uk/ http://pharma.bayer.com/ | 26/04/2016 |
|  | Biogen Idec (AVONEX and PLEGRIDY) | https://www.biogen-international.com/ https://www.biogen.uk.com/ | 28/04/2016 |
|  | Merck Serono (REBIF) | http://biopharma.merckgroup.com/en/index.html | 28/04/2016 |
|  | Novartis (EXTAVIA) | https://www.novartis.com https://www.novartis.co.uk/ | 28/04/2016 |
|  | Teva Pharmaceuticals (COPAXONE) | http://www.tevapharm.com/research_development/ http://www.tevauk.com/ | 01/05/2016 |
| **Patient carer groups** | Brain and Spine Foundation | http://www.brainandspine.org.uk | 01/05/2016 |
|  | Multiple Sclerosis National Therapy Centres | http://www.msntc.org.uk | 01/05/2016 |
|  | MS UK | http://www.ms-uk.org | 01/05/2016 |
|  | Multiple Sclerosis Society | https://www.mssociety.org.uk | 01/05/2016 |
|  | Multiple Sclerosis Trust | https://www.mstrust.org.uk | 01/05/2016 |
|  | Neurological Alliance | http://www.neural.org.uk | 01/05/2016 |
|  | The Brain Charity (formally known as Neurosupport) | http://www.thebraincharity.org.uk | 01/05/2016 |
|  | Sue Ryder | http://www.sueryder.org | 01/05/2016 |
| **Professional groups** | Association of British Neurologists | http://www.theabn.org | 01/05/2016 |
|  | British Neuropathological Society | http://www.bns.org.uk | 01/05/2016 |
|  | Institute of Neurology | https://www.ucl.ac.uk/ion https://www.ucl.ac.uk/ion/departments/neuroinflammation http://discovery.ucl.ac.uk | 01/05/2016 05/05/2016 10/05/2016 |
|  | Primary Care Neurology Society | http://www.p-cns.org.uk | 01/05/2016 |
|  | Therapists in MS | https://www.mstrust.org.uk/health-professionals/professional-networks/therapists-ms-tims/research | 01/05/2016 |
|  | UK Multiple Sclerosis Specialist Nurse Association | http://www.ukmssna.org.uk | 01/05/2016 |
| **Research groups** | Brain Research Trust | http://www.brt.org.uk/research | 01/05/2016 |
|  | British Neurological Research Trust | http://www.ukscf.org http://www.ukscf.org/about-us/bnrt.html | 01/05/2016 |
|  | Cochrane Multiple Sclerosis and Rare Diseases of the Central Nervous System | http://www.cochranelibrary.com http://msrdcns.cochrane.org/our-reviews | 01/05/2016 |
|  | National Institute for Health Research | http://www.nihr.ac.uk/research/ http://www.nihr.ac.uk/industry/ http://www.nihr.ac.uk/policy-and-standards/ | 01/05/2016 |

**Appendix S2. Data extraction**

Study acronym/ID:

**Name of the reviewer:**

**Number of publications extracted:**

| **Study details** |
| --- |
| Study ID (Endnote):  First author surname:  Year of publication:  Country:  Study setting:  Number of centres:  Study period:  Follow up period:  Funding:  Subtypes of MS included:  Definition of CIS used: |
| **Aim of the study** |
|  |
| **Participants** |
| Inclusion criteria:  Exclusion criteria:  Total number of participants:  Sample attrition/drop out:  Number of participants analysed:  **Characteristics of participants**  *Mean age:*  *Mean sex:*  *Race:*  *EDSS score at baseline:*  *Relapse rate at baseline:*  *Time from diagnosis of MS:*  *Other clinical features of MS:* |
| **Intervention (repeat if necessary for multiple intervention arms)** |
| Type of drug:  Method of administration:  Dose:  Frequency:  Drug indication as stated: |
| **Best supportive care as described** |
|  |
| **Outcomes** |
| Primary outcomes:  Secondary outcomes:  Method of assessing outcomes:  If freedom from disease activity is an outcome, how was it defined?:  Timing of assessment:  Adverse event:  Health related quality of life: Yes/No; which measures used? |

| **Number of participants** | **Intervention** | **Comparator, if present** |
| --- | --- | --- |
| Screened |  | |
| Excluded |  | |
| Randomised/Included |  |  |
| Missing participants (people who LTFU during the trial) |  |  |
| Withdrawals (all who did not complete, including LTFU) |  |  |
| **Patient baseline characteristics** | **Intervention:** | **Comparator:** |
| Age (years) |  |  |
| Sex |  |  |
| Race |  |  |
| EDSS score at baseline |  |  |
| Relapse rate at baseline |  |  |
| Time from diagnosis of MS |  |  |
| **Outcome data: relapses, disability** | **Intervention** | **Comparator, if present** |
| Relapse rate |  |  |
| Severity of relapse |  |  |
| Disability, including as measured by the Expanded Disability Status Scale |  |  |
| Freedom from disease activity |  |  |
| **Outcome data: MS symptoms (add rows as necessary)** | **Intervention** | **Comparator, if present** |
| Fatigue |  |  |
| Visual disturbance |  |  |
| Cognition |  |  |
| **Outcome data: additional outcomes** | **Intervention** | **Comparator, if present** |
| Mortality |  |  |
| Health-related quality of life |  |  |
| Progression to MS (CIS only) |  |  |
| Discontinuation due to neutralising antibody formation |  |  |
| **Adverse events (add rows as necessary for AEs reported in RCTs)** | **Intervention** | **Comparator, if present** |
|  |  |  |

**Risk of bias assessment**

| Random sequence generation | HIGH RISK UNCLEAR LOW RISK |
| --- | --- |
| *Description in trial* |  |
| Allocation concealment | HIGH RISK UNCLEAR LOW RISK |
| *Description in trial* |  |
| Blinding of participants and personnel | HIGH RISK UNCLEAR LOW RISK |
| *Description in trial* |  |
| Blinding of outcome assessment | HIGH RISK UNCLEAR LOW RISK |
| *Description in trial* |  |
| Incomplete outcome data | HIGH RISK UNCLEAR LOW RISK |
| *Description in trial* |  |
| Selective reporting | HIGH RISK UNCLEAR LOW RISK |
| *Description in trial* |  |
| Other sources of bias | HIGH RISK UNCLEAR LOW RISK |
| *Description in trial* |  |

| **Authors conclusion** |
| --- |
|  |
| **Reviewer’s conclusion** |
|  |

**Appendix S3. Data preparation**

Many of the included studies did not present adequate data for key findings to enable inclusion prima facie in a meta-analysis model. We used a variety of published methods to derive the necessary data.

Across all studies, we used data for the point of greatest maturity (i.e., last available follow-up) for which effect sizes were estimable. In studies presenting estimates with confirmed relapses and with non-confirmed relapses, we selected estimates with confirmed relapses.

We used rate ratios to examine relapse outcomes (e.g. the ratio of annualised relapse rates in two study arms). Other measures, such as time to relapse and proportion relapse-free, were inconsistently presented and at times impossible to impute, and proportion relapse-free would have been especially dependent on duration of follow-up and would not have captured the impact of drugs on multiple relapses per person. We used summary statistics instead of attempting to approximate individual participant data for each arm, in part due to the use of stratification in estimating study findings. Where necessary, we imputed standard errors by estimating the number of events in each arm (e.g. when relapse rates were analysed using an analysis of variance, or ANOVA, model with Gaussian link, instead of the preferred Poisson distribution for count variables). When arm-level annualised relapse rates (ARRs) were presented without Poisson-based standard errors, we generally assumed that the ARR presented for study arms was a fair approximation and then re-estimated the standard errors for the rate ratio using all available information on person-years of follow-up and number of relapses. Rate ratios were then analysed using a lognormal distribution.

We used hazard ratios to examine time to event outcomes (e.g. time to first relapse or time to confirmed disability progression). Other reported outcomes, such as proportions of patients with disability progression and magnitude of EDSS change, would have been especially dependent on duration of follow-up; in particular, data for magnitude of EDSS change would have required extensive imputation. Thus, we did not consider these. Where hazard ratios were not estimated from a Cox proportional hazards model, we used several methods in order of priority. First, we used methods published by Tierney et al. (2007) to estimate the HR, in particular using the number of patients analysed, the number of total events and the p-value derived from a log-rank test. When those data were not available to us, we then used the final predicted probabilities of survival in each study arm (generally estimated using Kaplan-Meier curves) and estimated the cumulative hazard using the equation –ln(S(t)), where S(t) is the probability of survival at time t. We then took the ratio of the cumulative hazards and used the log-rank p-value to approximate the standard errors for the HR, under the property that the p-value from the log-rank test for survival asymptotically approaches the p-value from a likelihood ratio test derived from a Cox proportional hazards model.

Finally, we used risk ratios to examine discontinuation due to AEs. In order to estimate these models, we examined three outcomes as reported: discontinuation of study drug due to AEs, discontinuation of study due to AEs, and withdrawal from study due to AEs. In the few studies that reported both discontinuation of study drug due to AEs and discontinuation of study due to AEs, we chose discontinuation of study drug due to AEs as we believed it would be a closer match to capturing the relationship between study drugs and discontinuation.
