# Supplementary material for: Comparative effectiveness of beta-interferons and glatiramer acetate for relapsing-remitting multiple sclerosis: systematic review and network meta-analysis of trials including recommended dosages
Source: BMC Neurol. 2018 Oct 3;18:162. doi: 10.1186/s12883-018-1162-9 (PMC6169084; doi:10.1186/s12883-018-1162-9)
Supplement: Supplementary file 2 — Additional results. This file includes detailed reasons for exclusion, tables of included publications, and sensitivity analyses for ARR, and detailed findings for discontinuation due to adverse events. (DOCX 265 kb) [file 12883_2018_1162_MOESM2_ESM.docx]

**Additional file 2: additional results**

**Table S1.** Frequency of reasons for study exclusion in the clinical effectiveness review

**Table S2.** Studies included in the clinical effectiveness review with relevant publications

**Table 3.** Network meta-analysis results for annualised relapse rate excluding Bornstein 1987.

**Figure S1.** Discontinuation due to adverse events: 24-month follow-up.

**Figure S2.** Discontinuation due to adverse events: all studies.

**Table S4.** Network meta-analysis results for discontinuation due to adverse events at 24-month follow-up.

**Table S5.** Network meta-analysis results for discontinuation due to adverse events at longest follow-up.

Table S1. Frequency of reasons for study exclusion in the clinical effectiveness review

| **Reasons** | **Number** |
| --- | --- |
| Conference abstract | 10 |
| DMT used with a non-recommended dose regimen | 15 |
| Irrelevant comparator/ intervention | 58 |
| Irrelevant comparator/ intervention/outcome | 1 |
| Irrelevant comparator/ intervention/population | 1 |
| Irrelevant comparator/ intervention/ study type | 4 |
| Irrelevant comparator/population | 5 |
| Irrelevant comparator/population/study type | 1 |
| Irrelevant intervention | 7 |
| Irrelevant intervention/population | 2 |
| Irrelevant intervention/ study type | 8 |
| Irrelevant outcome | 16 |
| Irrelevant outcome/study type | 2 |
| Irrelevant outcome/study type/population | 1 |
| Irrelevant population | 29 |
| Irrelevant population/outcomes | 1 |
| Irrelevant population/study type | 7 |
| Irrelevant study type | 24 |
| No results are provided, refers to results from a conference abstract | 1 |
| Not a primary research study | 3 |
| Not English language | 1 |
| Protocol only with no results | 15 |
| Systematic reviews that didn’t enable to locate further primary studies | 18 |
| Study evaluating a treatment-switch strategy | 1 |
| Use of an unlicensed drug formulation | 1 |
| **Total** | **232** |

Table S2. Studies included in the clinical effectiveness review with relevant publications

| **Study ID** | **Title** | **Full article(s) – main** | **Full article(s) - other** |
| --- | --- | --- | --- |
| ADVANCE 2014 | A Multicenter, Randomized, Double-Blind, Parallel-Group, Placebo-Controlled Study to Evaluate the Efficacy and Safety of PEGylated Interferon Beta-1a (BIIB017) in Subjects With Relapsing Multiple Sclerosis | Calabresi 2014[1] | Arnold 2014[2] (MRI), Newsome 2015[3] (HRQoL) |
| AVANTAGE 2014 | Safety Study in Relapsing-remitting Multiple Sclerosis (RRMS) Patients Receiving Betaferon or Rebif | No formal publication, results on company website[4] and ClinicalTrials.gov |  |
| BEYOND 2009 | International, Randomized, Multicenter, Phase IIIb Study in Patients With Relapsing-Remitting Multiple Sclerosis Comparing Over a Treatment Period of at Least 104 Weeks: 1. Double-Blinded Safety, Tolerability, and Efficacy of Betaseron/ Betaferon 250 µg (8 MIU) and Betaseron/-Betaferon 500 µg (16 MIU), Both Given Subcutaneously Every Other Day, and 2. Rater-Blinded Safety, Tolerability, and Efficacy of Betaseron/-Betaferon s.c. Every Other Day With Copaxone 20 mg s.c. Once Daily. | O'Connor 2009[5] | Filippi 2011[6] (Post hoc analysis of MRI scans) |
| Bornstein 1987 | A pilot trial of Cop 1 in exacerbating-remitting multiple sclerosis | Bornstein 1987[7] |  |
| BRAVO 2014 | A Multinational, Multicenter, Randomized, Parallel-group Study Performed in Subjects With RRMS to Assess the Efficacy, Safety and Tolerability of Laquinimod Over Placebo in a Double-blind Design and a Reference Arm of Interferon β-1a (Avonex®) in a Rater-blinded Design. | Vollmer 2014[8] |  |
| Calabrese 2012 | Effect of disease-modifying drugs on cortical lesions and atrophy in relapsing–remitting multiple sclerosis | Calabrese 2012[9] |  |
| CombiRx 2013 | A Multi-Center, Double-Blind, Randomized Study Comparing the Combined Use of Interferon Beta-1a and Glatiramer Acetate to Either Agent Alone in Patients With Relapsing-Remitting Multiple Sclerosis (CombiRx) | Lublin 2013[10] |  |
| CONFIRM 2012 | A Randomized, Multicenter, Placebo-Controlled and Active Reference (Glatiramer Acetate) Comparison Study to Evaluate the Efficacy and Safety of BG00012 in Subjects With Relapsing-Remitting Multiple Sclerosis | Fox 2012[11] | Kita 2014[12] (HRQoL) |
| Cop1 MSSG 1995 |  | Johnson 1995[13] (initial findings) | Johnson 1998[14] (final results) |
| ECGASG 2001 | European/Canadian Multicenter, Double-Blind, Randomized, Placebo-Controlled Study of the Effects of Glatiramer Acetate on Magnetic Resonance Imaging–Measured Disease Activity and Burden in Patients with Relapsing Multiple Sclerosis | Comi 2001[15] |  |
| Etemadifar 2006 | Comparison of Betaferon, Avonex, and Rebif in treatment of relapsing–remitting multiple sclerosis | Etemadifar 2006[16] |  |
| EVIDENCE 2007 | Full Results of the Evidence of Interferon Dose-Response-European North American Comparative Efficacy (EVIDENCE) Study: A Muhicenter, Randomized, Assessor-Blinded Comparison of Low-Dose Weekly Versus High-Dose, High-Frequency Interferon 13-1a for Relapsing Multiple Sclerosis | Schwid 2007[17] | Panitch 2002[18] (comparative results), Panitch 2005[19] (final comparative results), Sandberg-Wollheim 2005[20] (AEs) |
| GALA 2013 | Three Times Weekly Glatiramer Acetate in Relapsing–Remitting Multiple Sclerosis | Khan 2013[21] |  |
| GATE 2015 | Multi-centre, Randomized, Double-blind, Placebo-controlled, Parallel-group, 9 Month, Equivalence Trial Comparing the Efficacy and Safety and Tolerability of GTR (Synthon BV) to Copaxone® (Teva) in Subjects With Relapsing Remitting Multiple Sclerosis Followed by an Open-label 15 Month GTR Treatment Part Evaluating the Long-term GTR Treatment Effects | Cohen 2015[22] |  |
| IFNB MSSG 1995 | Interferon beta-lb is effective in relapsing-remitting multiple sclerosis. I. Clinical results of a multicenter, randomized, double-blind, placebo-controlled trial | IFNB Multiple Sclerosis Study Group 1993[23] | IFNB Multiple Sclerosis Study Group 1995[24] (additional data and further details) |
| IMPROVE 2012 | A Two-arm, Randomized, Double-blind, Control Group-compared, Multicenter, Phase IIIb Study With Monthly MRI and Biomarker Assessments to Evaluate the Efficacy, Safety, and Tolerability of Rebif® New Formulation (IFN Beta-1a) in Subjects With Relapsing Remitting Multiple Sclerosis | De Stefano 2012[25] |  |
| INCOMIN 2001 | Every-other-day interferon beta-1b versus once-weekly interferon beta-1a for multiple sclerosis: results of a 2-year prospective randomised multicentre study (INCOMIN) | Durelli 2002[26] |  |
| Kappos 2011 | Phase II, Multicenter, Randomized, Parallel-Group, Partially Blinded, Placebo and Avonex Controlled Dose Finding Study to Evaluate the Efficacy As Measured by Brain MRI Lesions, and Safety of 2 Dose Regimens of Ocrelizumab in Patients With RRMS | Kappos 2011[27] |  |
| Knobler 1993 | Systemic Recombinant Human Interferon-ß Treatment of Relapsing-Remitting Multiple Sclerosis: Pilot Study Analysis and Six-Year Follow-Up | Knobler 1993[28] |  |
| MSCRG 1996 | Intramuscular Interferon Beta-la for Disease Progression in Relapsing Multiple Sclerosis | Jacobs 1996[29] | Fischer 2000,[30] Goodkin 1998,[31] Granger 2003,[32] Miller 2011,[33] Rudick 1997[34] |
| PRISMS 1998 | Randomised double-blind placebo-controlled study of interferon beta-1a in relapsing/remitting multiple sclerosis | PRISMS Study Group 1998[35] | Patten 2001[36] (depression), Gold 2005[37] (4 year safety and tolerability) |
| REFLEX 2012 | A Phase III, Randomized, Double-blind, Placebo-controlled, Multicenter Clinical Trial of Rebif New Formulation (44 Microgram [Mcg] Three Times Weekly [Tiw] and 44 Mcg Once Weekly [ow]) in Subjects at High Risk of Converting to Multiple Sclerosis (REFLEX) | Comi 2012[38] | Freedman 2014[39] (Subgroup analysis), CADTH 2013[40] |
| REFORMS 2012 | A Randomized, Multicenter, Two Arm, Open Label, Twelve Week Phase IIIb Study to Evaluate the Tolerability of Rebif (New Formulation) (IFN Beta-1a) and Betaseron (IFN Beta-1b) in IFN-naive Subjects With Relapsing Remitting Multiple Sclerosis (RRMS) Followed by a Single Arm, Eighty-two Week Minimum, Rebif (New Formulation) Only Safety Extension | Singer 2012[41] |  |
| REGARD 2008 | Phase IV, Multicenter, Open Label, Randomized Study of Rebif® 44 mcg Administered Three Times Per Week by Subcutaneous Injection Compared With Copaxone® 20 mg Administered Daily by Subcutaneous Injection in the Treatment of Relapsing Remitting Multiple Sclerosis | Mikol 2008[42] |  |

**References**

1. Calabresi PA, Kieseier BC, Arnold DL, Balcer LJ, Boyko A, Pelletier J et al. Pegylated interferon beta-1a for relapsing-remitting multiple sclerosis (ADVANCE): a randomised, phase 3, double-blind study. Lancet Neurol 2014, 13:657-65.

2. Arnold DL, Calabresi PA, Kieseier BC, Sheikh SI, Deykin A, Zhu Y et al. Effect of peginterferon beta-1a on MRI measures and achieving no evidence of disease activity: results from a randomized controlled trial in relapsing-remitting multiple sclerosis. BMC Neurol 2014, 14:240.

3. Newsome SD, Guo S, Altincatal A, Proskorovsky I, Kinter E, Phillips G et al. Impact of peginterferon beta-1a and disease factors on quality of life in multiple sclerosis. Mult Scler Relat Disord 2015, 4:350-7.

4. Clinical Study Synopsis: The AVANTAGE study - A randomized, multicenter, phase IV, open-label prospective study comparing injection site reaction and injection site pain in patients with relapsing remitting multiple sclerosis (RRMS) or after a first demyelinating event suggestive of MS newly started on interferon beta-1b (Betaferon®) or interferon beta-1a (Rebif®). Trial finder: Bayer HealthCare AG; 2013. URL: <http://trialfinder.pharma.bayer.com/omr/online/91489_Study_Synopsis_CTP.pdf>. Accessed 01 May 2016.

5. O'Connor P, Filippi M, Arnason B, Comi G, Cook S, Goodin D et al. 250 microg or 500 microg interferon beta-1b versus 20 mg glatiramer acetate in relapsing-remitting multiple sclerosis: a prospective, randomised, multicentre study. Lancet Neurol 2009, 8:889-97.

6. Filippi M, Rocca MA, Camesasca F, Cook S, O'Connor P, Arnason BG et al. Interferon ?-1b and glatiramer acetate effects on permanent black hole evolution. Neurology 2011, 76:1222-8.

7. Bornstein MB, Miller A, Slagle S, Weitzman M, Crystal H, Drexler E et al. A pilot trial of Cop 1 in exacerbating-remitting multiple sclerosis. N Engl J Med 1987, 317:408-14.

8. Vollmer TL, Sorensen PS, Selmaj K, Zipp F, Havrdova E, Cohen JA et al. A randomized placebo-controlled phase III trial of oral laquinimod for multiple sclerosis. J Neurol 2014, 261:773-83.

9. Calabrese M, Bernardi V, Atzori M, Mattisi I, Favaretto A, Rinaldi F et al. Effect of disease-modifying drugs on cortical lesions and atrophy in relapsing-remitting multiple sclerosis. Mult Scler 2012, 18:418-24.

10. Lublin FD, Cofield SS, Cutter GR, Conwit R, Narayana PA, Nelson F et al. Randomized study combining interferon and glatiramer acetate in multiple sclerosis. Ann Neurol 2013, 73:327-40.

11. Fox RJ, Miller DH, Phillips JT, Hutchinson M, Havrdova E, Kita M et al. Placebo-controlled phase 3 study of oral BG-12 or glatiramer in multiple sclerosis.[Erratum appears in N Engl J Med. 2012 Oct 25;367(17):1673]. N Engl J Med 2012, 367:1087-97.

12. Kita M, Fox RJ, Phillips JT, Hutchinson M, Havrdova E, Sarda SP et al. Effects of BG-12 (dimethyl fumarate) on health-related quality of life in patients with relapsing-remitting multiple sclerosis: findings from the CONFIRM study. Mult Scler 2014, 20:253-7.

13. Johnson KP, Brooks BR, Cohen JA, Ford CC, Goldstein J, Lisak RP et al. Copolymer 1 reduces relapse rate and improves disability in relapsing-remitting multiple sclerosis: results of a phase III multicenter, double-blind placebo-controlled trial. The Copolymer 1 Multiple Sclerosis Study Group. Neurology 1995, 45:1268-76.

14. Johnson KP, Brooks BR, Cohen JA, Ford CC, Goldstein J, Lisak RP et al. Extended use of glatiramer acetate (Copaxone) is well tolerated and maintains its clinical effect on multiple sclerosis relapse rate and degree of disability. Neurology 1998, 50:701-8.

15. Comi G, Filippi M, Wolinsky JS. European/Canadian multicenter, double-blind, randomized, placebo-controlled study of the effects of glatiramer acetate on magnetic resonance imaging-measured disease activity and burden in patients with relapsing multiple sclerosis. Ann Neurol 2001, 49:290-7.

16. Etemadifar M, Janghorbani M, Shaygannejad V. Comparison of Betaferon, Avonex, and Rebif in treatment of relapsing-remitting multiple sclerosis. Acta Neurol Scand 2006, 113:283-7.

17. Schwid SR, Panitch HS. Full results of the Evidence of Interferon Dose-Response-European North American Comparative Efficacy (EVIDENCE) study: a multicenter, randomized, assessor-blinded comparison of low-dose weekly versus high-dose, high-frequency interferon beta-1a for relapsing multiple sclerosis. Clin Ther 2007, 29:2031-48.

18. Panitch H, Goodin DS, Francis G, Chang P, Coyle PK, O’Connor P et al. Randomized, comparative study of interferon B-1a treatment regimens in MS: The EVIDENCE Trial. Neurology 2002, 59:1496–506.

19. Panitch H, Goodin DS, Francis G, Chang P, Coyle PK, O’Connor P et al. Benefits of high-dose, high-frequency interferon beta-1a in relapsing–remitting multiple sclerosis are sustained to 16 months: Final comparative results of the EVIDENCE trial. J Neurol Sci 2005, 239:67-74.

20. Sandberg-Wollheim M, Bever C, Carter J, Färkkilä M, Hurwitz B, Lapierre Y et al. Comparative tolerance of IFN beta-1a regimens in patients with relapsing multiple sclerosis: The EVIDENCE study. J Neurol 2005, 252:8-13.

21. Khan O, Rieckmann P, Boyko A, Selmaj K, Zivadinov R, Group GS. Three times weekly glatiramer acetate in relapsing-remitting multiple sclerosis. Ann Neurol 2013, 73:705-13.

22. Cohen J, Belova A, Selmaj K, Wolf C, Sormani MP, Oberye J et al. Equivalence of Generic Glatiramer Acetate in Multiple Sclerosis: A Randomized Clinical Trial. JAMA Neurol 2015, 72:1433-41.

23. IFNB Multiple Sclerosis Study Group. Interferon beta-1b is effective in relapsing-remitting multiple sclerosis. I. Clinical results of a multicenter, randomized, double-blind, placebo-controlled trial. The IFNB Multiple Sclerosis Study Group. Neurology 1993, 43:655-61.

24. IFNB Multiple Sclerosis Study Group, University of British Columbia MS/MRI Analysis Group. Interferon beta-lb in the treatment of multiple sclerosis: Final outcome of the randomized controlled trial. Neurology 1995, 45:1277-85.

25. De Stefano N, Sormani MP, Stubinski B, Blevins G, Drulovic JS, Issard D et al. Efficacy and safety of subcutaneous interferon B-1a in relapsing-remitting multiple sclerosis: further outcomes from the IMPROVE study. J Neurol Sci 2012, 312:97-101.

26. Durelli L, Verdun E, Barbero P, Bergui M, Versino E, Ghezzi A et al. Every-other-day interferon beta-1b versus once-weekly interferon beta-1a for multiple sclerosis: results of a 2-year prospective randomised multicentre study (INCOMIN). Lancet 2002, 359:1453-60.

27. Kappos L, Li D, Calabresi PA, O'Connor P, Bar-Or A, Barkhof F et al. Ocrelizumab in relapsing-remitting multiple sclerosis: a phase 2, randomised, placebo-controlled, multicentre trial. Lancet 2011, 378:1779-87.

28. Knobler RL, Greenstein JI, Johnson KP, Lublin FD, Panitch HS, Conway K et al. Systemic recombinant human interferon-beta treatment of relapsing-remitting multiple sclerosis: pilot study analysis and six-year follow-up. J Interferon Res 1993, 13:333-40.

29. Jacobs LD, Cookfair DL, Rudick RA, Herndon RM, Richert JR, Salazar AM et al. Intramuscular interferon beta-1a for disease progression in relapsing multiple sclerosis. The Multiple Sclerosis Collaborative Research Group (MSCRG). Ann Neurol 1996, 39:285-94.

30. Fischer JS, Priore RL, Jacobs LD, Cookfair DL, Rudick RA, Herndon RM et al. Neuropsychological effects of interferon beta-1a in relapsing multiple sclerosis. Multiple Sclerosis Collaborative Research Group. Ann Neurol 2000, 48:885-92.

31. Goodkin DE, Priore RL, Wende KE, Campion M, Bourdette DN, Herndon RM et al. Comparing the ability of various compositive outcomes to discriminate treatment effects in MS clinical trials. The Multiple Sclerosis Collaborative Research Group (MSCRG). Mult Scler 1998, 4:480-6.

32. Granger C, Wende K, Brownscheidle C. Use of the FIM™ Instrument in a Trial of Intramuscular Interferon B-1a for Disease Progression in Relapsing-Remitting Multiple Sclerosis. Am J Phys Med Rehabil 2003, 82:427-36.

33. Miller DM, Weinstock-Guttman B, Bourdette D, You X, Foulds P, Rudick RA. Change in quality of life in patients with relapsing-remitting multiple sclerosis over 2 years in relation to other clinical parameters: results from a trial of intramuscular interferon {beta}-1a. Mult Scler 2011, 17:734-42.

34. Rudick RA, Goodkin DE, Jacobs LD, Cookfair DL, Herndon RM, Richert JR et al. Impact of interferon beta-1a on neurologic disability in relapsing multiple sclerosis. The Multiple Sclerosis Collaborative Research Group (MSCRG). Neurology 1997, 49:358-63.

35. PRISMS Study Group. Randomised double-blind placebo-controlled study of interferon beta-1a in relapsing/remitting multiple sclerosis. PRISMS (Prevention of Relapses and Disability by Interferon beta-1a Subcutaneously in Multiple Sclerosis) Study Group. Lancet 1998, 352:1498-504.

36. Patten SB, Metz LM. Interferon beta-1 a and depression in relapsing-remitting multiple sclerosis: an analysis of depression data from the PRISMS clinical trial. Mult Scler 2001, 7:243-8.

37. Gold R, Rieckmann P, Chang P, Abdalla J. The long-term safety and tolerability of high-dose interferon beta-1a in relapsing-remitting multiple sclerosis: 4-year data from the PRISMS study. Eur J Neurol 2005, 12:649-56.

38. Comi G, De Stefano N, Freedman MS, Barkhof F, Polman CH, Uitdehaag BMJ et al. Comparison of two dosing frequencies of subcutaneous interferon beta-1a in patients with a first clinical demyelinating event suggestive of multiple sclerosis (REFLEX): A phase 3 randomised controlled trial. Lancet Neurol 2012, 11:33-41.

39. Freedman MS, De Stefano N, Barkhof F, Polman CH, Comi G, Uitdehaag BM et al. Patient subgroup analyses of the treatment effect of subcutaneous interferon beta-1a on development of multiple sclerosis in the randomized controlled REFLEX study. J Neurol 2014, 261:490-9.

40. Common Drug Review: CDEC FINAL RECOMMENDATION: INTERFERON BETA-1A (Rebif - EMD Serono Canada Inc.) Indication: Clinically Isolated Syndrome. Canadian Agency for Drugs and Technologies in Health (CADTH); 2013. URL: <https://www.cadth.ca/media/cdr/complete/cdr_complete_Rebif_Aug-19-13.pdf>. Accessed 01/06/2016.

41. Singer B, Bandari D, Cascione M, LaGanke C, Huddlestone J, Bennett R et al. Comparative injection-site pain and tolerability of subcutaneous serum-free formulation of interferonbeta-1a versus subcutaneous interferonbeta-1b: results of the randomized, multicenter, Phase IIIb REFORMS study. BMC Neurol 2012, 12:154.

42. Mikol DD, Barkhof F, Chang P, Coyle PK, Jeffery DR, Schwid SR et al. Comparison of subcutaneous interferon beta-1a with glatiramer acetate in patients with relapsing multiple sclerosis (the REbif vs Glatiramer Acetate in Relapsing MS Disease [REGARD] study): a multicentre, randomised, parallel, open-label trial. Lancet Neurol 2008, 7:903-14.

**Table S3.** Network meta-analysis results for annualised relapse rate excluding Bornstein 1987.*

| **Drug** | **SUCRA** | **PegIFN β-1a 125 μg every 2 weeks** | **Glatiramer 40 mg thrice weekly** | **Glatiramer 20 mg daily** | **IFN β-1a 44 μg SC thrice weekly** | **IFN β-1b 250 μg SC every other day** | **IFN β-1a 22 μg SC thrice weekly** | **IFN β-1a 30 μg IM weekly** | **Placebo** |
| --- | --- | --- | --- | --- | --- | --- | --- | --- | --- |
| PegIFN β-1a 125 μg every 2 weeks | 0.76 |  | 0.98 (0.71, 1.35) | 0.95 (0.73, 1.25) | 0.94 (0.71, 1.24) | 0.92 (0.70, 1.21) | 0.89 (0.66, 1.20) | 0.80 (0.61, 1.05) | 0.64 (0.50, 0.83) |
| Glatiramer 40 mg thrice weekly | 0.73 |  |  | 0.97 (0.78, 1.21) | 0.96 (0.77, 1.20) | 0.94 (0.75, 1.17) | 0.91 (0.70, 1.17) | 0.82 (0.65, 1.02) | 0.66 (0.54, 0.80) |
| Glatiramer 20 mg daily | 0.69 |  |  |  | 0.99 (0.87, 1.12) | 0.98 (0.86, 1.12) | 0.93 (0.78, 1.12) | 0.84 (0.74, 0.95) | 0.68 (0.61, 0.75) |
| IFN β-1a 44 μg SC thrice weekly | 0.65 |  |  |  |  | 0.98 (0.86, 1.12) | 0.94 (0.80, 1.11) | 0.85 (0.76, 0.95) | 0.68 (0.61, 0.76) |
| IFN β-1b 250 μg SC every other day | 0.55 |  |  |  |  |  | 0.96 (0.80, 1.15) | 0.87 (0.77, 0.98) | 0.70 (0.63, 0.77) |
| IFN β-1a 22 μg SC thrice weekly | 0.45 |  |  |  |  |  |  | 0.90 (0.76, 1.07) | 0.72 (0.62, 0.85) |
| IFN β-1a 30 μg IM weekly | 0.17 |  |  |  |  |  |  |  | 0.80 (0.73, 0.89) |
| Placebo | 0.00 |  |  |  |  |  |  |  |  |
| Wald test for inconsistency (χ2, df, p) | | 12.59, 11, 0.32 |  |  |  |  |  |  |  |

*Findings are expressed as rate ratio (RR) with 95% CI.

**Figure S1.** Discontinuation due to adverse events: 24-month follow-up.


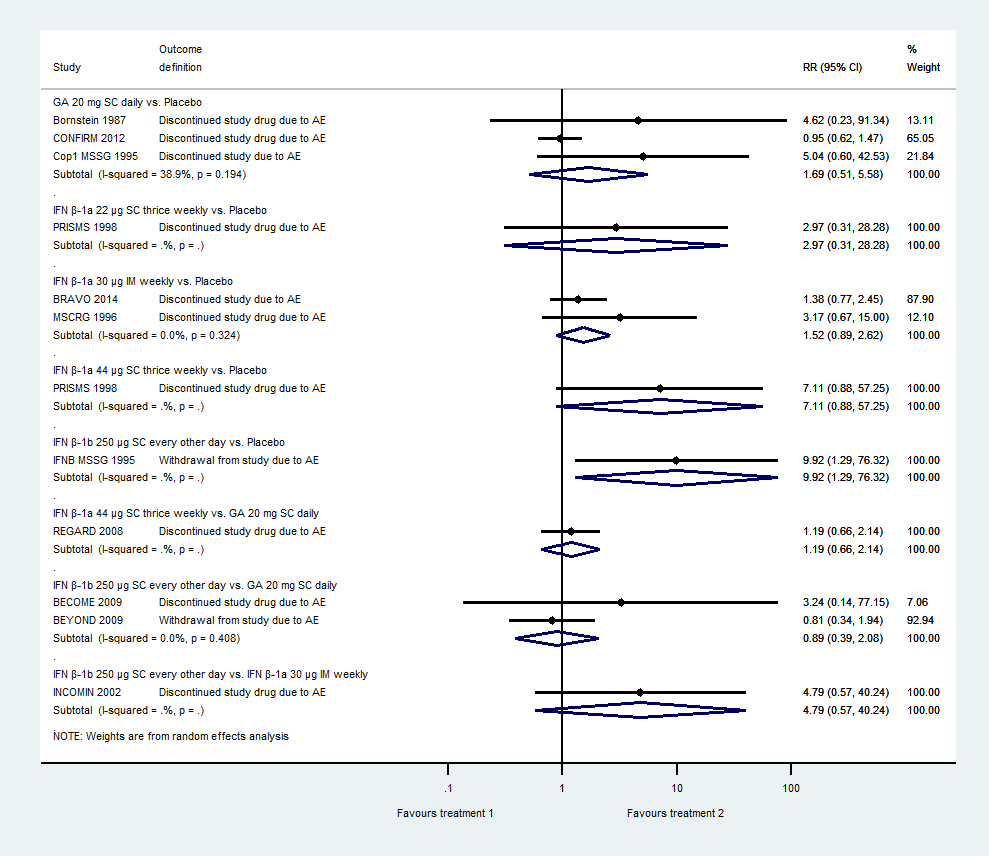


**Figure S2.** Discontinuation due to adverse events: all studies.


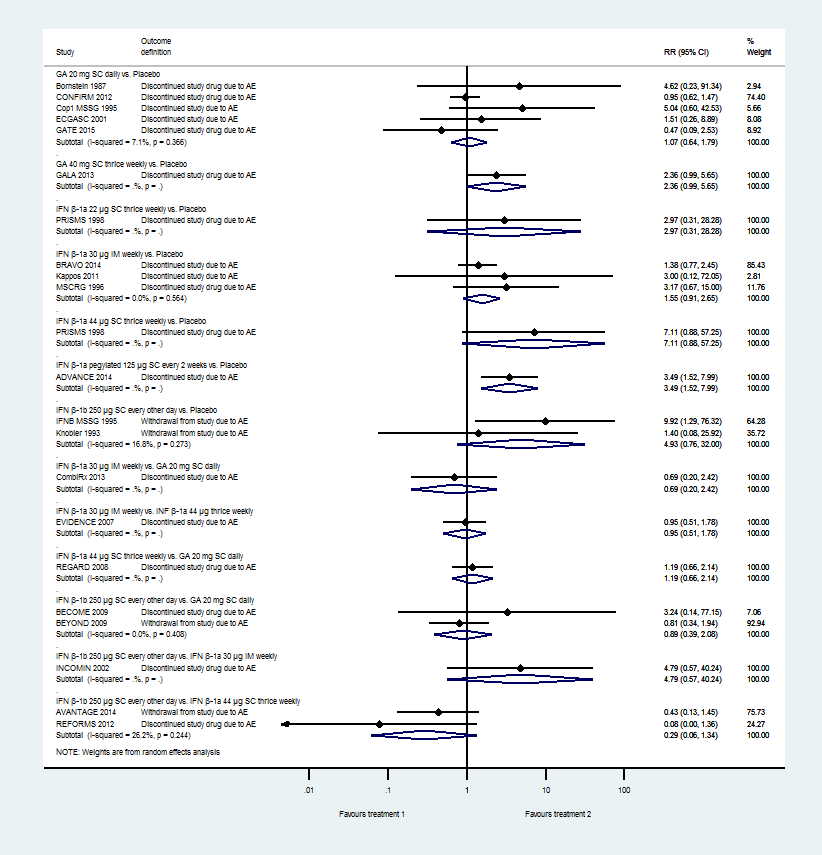


**Table S4.** Network meta-analysis results for discontinuation due to adverse events at 24-month follow-up.*

| **Drug** | **SUCRA** | IFN β-1b 250 μg SC every other day | IFN β-1a 44 μg SC thrice weekly | GA 20 mg daily | IFN β-1a 22 μg SC thrice weekly | IFN β-1a 30 μg IM weekly | Placebo |
| --- | --- | --- | --- | --- | --- | --- | --- |
| IFN β-1b 250 μg SC every other day | 0.79 |  | 1.15 (0.20, 6.56) | 1.70 (0.50, 5.81) | 2.37 (0.22, 25.84) | 2.74 (0.56, 13.38) | 4.41 (1.07, 18.29) |
| IFN β-1a 44 μg SC thrice weekly | 0.76 |  |  | 1.48 (0.39, 5.57) | 2.07 (0.32, 13.44) | 2.39 (0.38, 15.22) | 3.85 (0.81, 18.29) |
| GA 20 mg daily | 0.57 |  |  |  | 1.40 (0.17, 11.76) | 1.61 (0.38, 6.91) | 2.60 (0.88, 7.64) |
| IFN β-1a 22 μg SC thrice weekly | 0.41 |  |  |  |  | 1.15 (0.10, 13.09) | 1.86 (0.21, 16.83) |
| IFN β-1a 30 μg IM weekly | 0.35 |  |  |  |  |  | 1.61 (0.52, 5.02) |
| Placebo | 0.12 |  |  |  |  |  |  |
| Wald test for inconsistency (χ2, df, p) | | 2.38, 3, 0.50 |  |  |  |  |  |

*Results are presented as risk ratio (95% CI).

**Table S5.** Network meta-analysis results for discontinuation due to adverse events at longest follow-up.*

| **Drug** | **SUCRA** | PegIFN β-1a 125 μg every 2 weeks | IFN β-1a 44 μg SC thrice weekly | GA 40 mg thrice weekly | IFN β-1b 250 μg SC every other day | IFN β-1a 30 μg IM weekly | GA 20 mg daily | IFN β-1a 22 μg SC thrice weekly | Placebo |
| --- | --- | --- | --- | --- | --- | --- | --- | --- | --- |
| PegIFN β-1a 125 μg every 2 weeks | 0.82 |  | 1.40 (0.31, 6.45) | 1.48 (0.29, 7.43) | 1.99 (0.43, 9.15) | 2.15 (0.57, 8.04) | 2.24 (0.59, 8.44) | 2.82 (0.35, 23.04) | 3.49 (1.13, 10.76) |
| IFN β-1a 44 μg SC thrice weekly | 0.73 |  |  | 1.05 (0.22, 4.95) | 1.42 (0.61, 3.30) | 1.53 (0.65, 3.59) | 1.60 (0.76, 3.36) | 2.01 (0.45, 9.01) | 2.49 (0.89, 6.95) |
| GA 40 mg thrice weekly | 0.66 |  |  |  | 1.35 (0.29, 6.35) | 1.45 (0.38, 5.60) | 1.52 (0.39, 5.89) | 1.91 (0.23, 15.88) | 2.36 (0.74, 7.53) |
| IFN β-1b 250 μg SC every other day | 0.50 |  |  |  |  | 1.08 (0.42, 2.79) | 1.12 (0.51, 2.49) | 1.42 (0.26, 7.71) | 1.75 (0.63, 4.89) |
| IFN β-1a 30 μg IM weekly | 0.45 |  |  |  |  |  | 1.04 (0.51, 2.13) | 1.32 (0.24, 7.17) | 1.62 (0.82, 3.23) |
| GA 20 mg daily | 0.40 |  |  |  |  |  |  | 1.26 (0.24, 6.50) | 1.56 (0.77, 3.14) |
| IFN β-1a 22 μg SC thrice weekly | 0.33 |  |  |  |  |  |  |  | 1.24 (0.21, 7.26) |
| Placebo | 0.12 |  |  |  |  |  |  |  |  |
| Wald test for inconsistency (χ2, df, p) | | 11.04, 6, 0.09 |  |  |  |  |  |  |  |

*Results are presented as risk ratio (95% CI).
